# Comprehensive analysis of the HEPN superfamily: identification of novel roles in intra-genomic conflicts, defense, pathogenesis and RNA processing

**DOI:** 10.1186/1745-6150-8-15

**Published:** 2013-06-15

**Authors:** Vivek Anantharaman, Kira S Makarova, A Maxwell Burroughs, Eugene V Koonin, L Aravind

**Affiliations:** 1 National Center for Biotechnology Information, National Library of Medicine, National Institutes of Health, Bethesda, MD 20894, USA

## Abstract

**Background:**

The major role of enzymatic toxins that target nucleic acids in biological conflicts at all levels has become increasingly apparent thanks in large part to the advances of comparative genomics. Typically, toxins evolve rapidly hampering the identification of these proteins by sequence analysis. Here we analyze an unexpectedly widespread superfamily of toxin domains most of which possess RNase activity.

**Results:**

The HEPN superfamily is comprised of all α-helical domains that were first identified as being associated with DNA polymerase β-type nucleotidyltransferases in prokaryotes and animal Sacsin proteins. Using sensitive sequence and structure comparison methods, we vastly extend the HEPN superfamily by identifying numerous novel families and by detecting diverged HEPN domains in several known protein families. The new HEPN families include the RNase LS and LsoA catalytic domains, KEN domains (e.g. RNaseL and Ire1) and the RNase domains of RloC and PrrC. The majority of HEPN domains contain conserved motifs that constitute a metal-independent endoRNase active site. Some HEPN domains lacking this motif probably function as non-catalytic RNA-binding domains, such as in the case of the mannitol repressor MtlR. Our analysis shows that HEPN domains function as toxins that are shared by numerous systems implicated in intra-genomic, inter-genomic and intra-organismal conflicts across the three domains of cellular life. In prokaryotes HEPN domains are essential components of numerous toxin-antitoxin (TA) and abortive infection (Abi) systems and in addition are tightly associated with many restriction-modification (R-M) and CRISPR-Cas systems, and occasionally with other defense systems such as Pgl and Ter. We present evidence of multiple modes of action of HEPN domains in these systems, which include direct attack on viral RNAs (e.g. LsoA and RNase LS) in conjunction with other RNase domains (e.g. a novel RNase H fold domain, NamA), suicidal or dormancy-inducing attack on self RNAs (RM systems and possibly CRISPR-Cas systems), and suicidal attack coupled with direct interaction with phage components (Abi systems). These findings are compatible with the hypothesis on coupling of pathogen-targeting (immunity) and self-directed (programmed cell death and dormancy induction) responses in the evolution of robust antiviral strategies. We propose that altruistic cell suicide mediated by HEPN domains and other functionally similar RNases was essential for the evolution of kin and group selection and cell cooperation. HEPN domains were repeatedly acquired by eukaryotes and incorporated into several core functions such as endonucleolytic processing of the 5.8S-25S/28S rRNA precursor (Las1), a novel ER membrane-associated RNA degradation system (C6orf70), sensing of unprocessed transcripts at the nuclear periphery (Swt1). Multiple lines of evidence suggest that, similar to prokaryotes, HEPN proteins were recruited to antiviral, antitransposon, apoptotic systems or RNA-level response to unfolded proteins (Sacsin and KEN domains) in several groups of eukaryotes.

**Conclusions:**

Extensive sequence and structure comparisons reveal unexpectedly broad presence of the HEPN domain in an enormous variety of defense and stress response systems across the tree of life. In addition, HEPN domains have been recruited to perform essential functions, in particular in eukaryotic rRNA processing. These findings are expected to stimulate experiments that could shed light on diverse cellular processes across the three domains of life.

**Reviewers:**

This article was reviewed by Martijn Huynen, Igor Zhulin and Nick Grishin

## Background

Over the past decade it has become increasingly evident that the deployment of enzymatic toxins that target nucleic acids is a common feature of biological conflicts at all levels [1-5]. These enzymes disrupt nucleic acids by cleaving their backbones, breaking glycosidic linkages between sugars and bases, or modifying bases. Among these enzymes, RNases that target tRNAs, rRNAs and mRNAs are among the most common toxins in various intra-genomic, intergenomic, and inter-organismal conflict systems [2,6-8]. In the case of intra-genomic selfish elements, the toxin component of the extremely abundant prokaryotic toxin-antitoxin (TA) systems most often are RNases, predominantly of RelE-like and PIN superfamilies [7,9-12]. In eukaryotes RNases are major contributors to the elaborate strategies of defense against intra-genomic selfish elements (transposons) [13-15]. This system specifically targets the selfish elements by means of Piwi RNases guided by piRNAs [16,17]. In prokaryotes, RNases are also represented among the toxin domains of various colicin-type bacteriocins, which are involved in inter-genomic conflicts between plasmids and cellular genomes [8,18,19].

Another common class of inter-genomic conflicts is that between viruses and the host cell genome [1]. In these conflicts the host cell often deploys toxin RNases to either cleave viral RNAs or target self RNAs to induce a dormancy or apoptotic response to limit viral replication and infection [4]. Such defense RNases encompass a wide range of proteins, such as the Abi system components, the interferon-induced RNase L in eukaryotes [20], and probably the RNases associated with the CRISPR-Cas adaptive immunity systems in prokaryotes [4,21,22]. In addition, recent studies have shown that secreted toxin RNases are extensively used in inter-organismal conflicts [8]. These include many of the toxin tips of the polymorphic toxins employed in intra-species conflicts, the fungal killer toxins and effectors deployed by bacteria against hosts or competitors [2,23,24]. Such RNase toxins are also part of the defense repertoire of multicellular forms, such as snake venoms [25] or factors that prevent self-fertilization in plants [26,27].

Recent studies indicate that many of the RNases related to those that participate in biological conflicts are involved in core cellular functions as RNA-processing enzymes. A case in point is the EndoU RNase domain that apparently was derived from ancestral RNases found in polymorphic toxins and related systems [2]. Upon acquisition by eukaryotes, this domain was recruited for splicing of intron-encoded U16 and U86 snoRNAs [28-30], and subsequently acquired by nidoviruses where it plays a role in RNA processing during replication [28]. Likewise, RNase L also functions as a splicing factor and a RNA-level regulator of the unfolded protein response in eukaryotes [20,31]. Other than their role as toxins in prokaryotic TA systems, distinct versions of the PIN domain also function as RNA-processing enzymes [32-34]. In particular, PIN domains comprise the active moieties of the RNases that target mRNAs with stop codons in the eukaryotic nonsense-mediate decay system [35,36]. The Piwi-Argonaute (Ago)-like RNaseH fold proteins, that are the key components of the eukaryotic RNAi response [37] and are implicated in defense in prokaryotes as well [38], also perform core cellular functions, especially in eukaryotes, in utilizing small RNAs to mediate chromatin condensation as part of gene silencing, chromosomal reorganization in the ciliate macronuclei, and post-transcriptional regulation of gene expression [39,40]. Thus, the study of RNase domains involved in biological conflicts also often throws light on the functions and molecular mechanisms of RNases participating in core cellular processes.

Our previous work has shown that investigation of proteinaceous toxins using sensitive sequence analysis and structure comparison techniques, combined with contextual information derived from genome comparisons, has considerable potential for discovery of new RNA-targeting activities [2,10,35]. Here we apply such computational methods to unravel the biochemistry and biology of an enigmatic domain, the so called HEPN (Higher Eukaryotes and Prokaryotes Nucleotide-binding domain) domain [41]. Initially, the HEPN domain was identified in proteins encoded by genes that, in bacteria and archaea, strictly co-localize with genes encoding minimal nucleotidyltransferases (MNTs) that belong to the DNA polymerase β-like protein superfamily [41,42]. This strict association led to the suggestion that HEPN domains functioned in conjunction with the associated MNT domains. The structural relationship of the HEPN domain with the substrate-binding domain of several polymerase β superfamily enzymes (e.g. kanamycin nucleotidyltransferase), whose nucleotidyltransferase domains are homologous to the MNT domain, led to the idea that HEPN might constitute the substrate-binding subunit of the MNTs [41]. In addition, distinct versions of the HEPN domain were found independent of the MNT gene-neighborhoods in association with some other domains, such as the HSP90-S5 fold domains in the human protein Sacsin [41]. The two component MNT-HEPN module has been predicted to function as a type II TA system, with MNT that appeared to be the only active enzyme in the system predicted to be the toxin and the HEPN domain the antitoxin [10]. A recent genome-wide screen for toxins has confirmed the TA function of the HEPN-MNT module, but contrary to the original prediction, identified the HEPN domain as the toxin in this system [43]. These findings prompted us to perform an exhaustive census and analysis of the HEPN domains in an attempt to better understand their toxicity, modes of action, spread in different organisms and evolution.

We describe here comprehensive sequence, structure and genomic context analyses that strongly support the interaction of the HEPN domain with nucleic acids in multiple systems involved in biological conflicts and processing of cellular RNAs. In particular, we present evidence that several diverse HEPN versions function as metal-independent RNases. Thus, the RNase activity of HEPN domain could be a unifying theme shared by cellular RNA maturation systems and those involved in biological conflicts.

## Results and discussion

### Sequence analysis of the HEPN superfamily and identification numerous novel families

**Figure 1:**
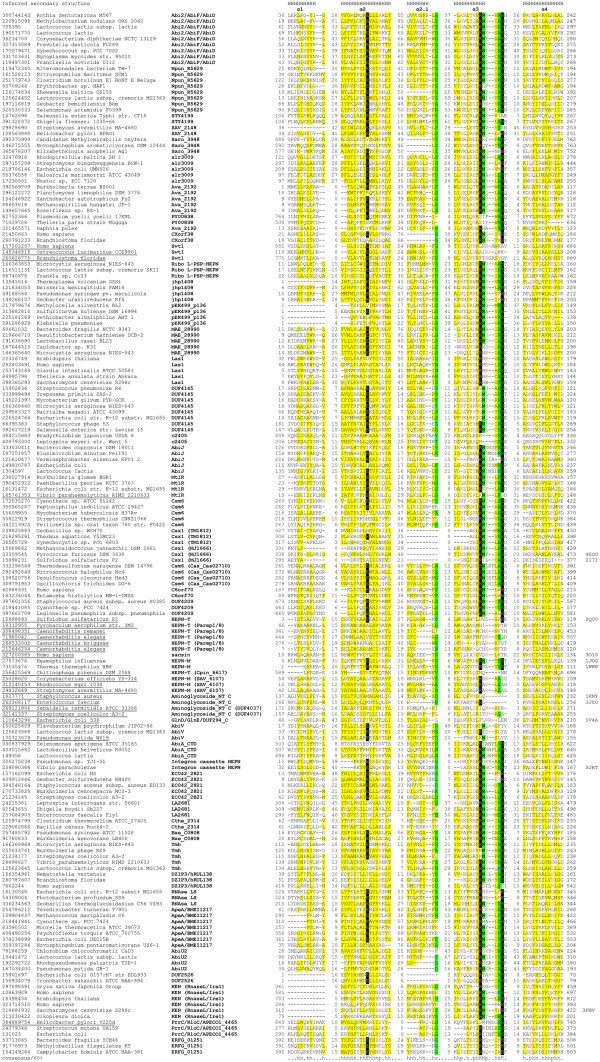
**Multiple alignment of the HEPN superfamily.** The multiple sequence alignment includes the conserved blocks based on the MUSCLE alignment [45], which was corrected manually on the basis of HHpred [46] and PSI-BLAST results [47]. Due to the low similarity, the alignment of helices 1, 2.1 and 4 should be considered tentative. Secondary structure, which is a consensus between the proteins with solved structures, is shown above the alignment; ‘H’ indicates α-helix. The sequences are denoted by their GI numbers and species names. The HEPN family to which each sequence belongs is indicated after the species name. Positions of the first and the last residues of the aligned region in the corresponding protein are indicated for each sequence. The PDB identifiers for proteins with solved structure are indicated on the right. The numbers (of amino acid residues) within the alignment represent poorly conserved inserts that are not shown. The coloring is based on the consensus shown underneath the alignment; ‘h’ indicates hydrophobic residues (WFYMLIVACTH), ‘p’ indicates polar residues (EDKRNQHTS),‘s’ indicates small residues (ACDGNPSTV). Predicted catalytic amino acids are shown by reverse shading. GI and species name is underlined if the HEPN domain has lost the conserved Rx4-6H motif.

Transitive, iterative sequence profile searches and hidden Markov model (HMM) searches with the originally defined HEPN domains [41,42] used as the queries using PSI-BLAST and HMM-SEARCH3 programs recovered an extended set of homologous domains. These included two families of so-called “domains of unknown function” from the Pfam database, namely DUF4145 and DUF86 all of which, along with models for the C-terminal domains of several polymerase β-superfamily proteins (Pfam models: GlnD_UR_UTase, NTase_sub_bind and DUF294_C), are currently included in the Pfam clan named CL0291. Of these, DUF86 includes proteins, most of which were originally reported as being encoded by genes adjacent to those for MNTs [41,42]. However, several representatives of DUF4145 are fused to restriction endonuclease (REase) and superfamily-II helicase modules, indicating that HEPN domains also commonly occur independently of MNTs. These iterative searches also recovered several borderline hits (e-values ~ 0.05-.2) which shared a conserved motif with the known HEPN domains (Figure 1, see below), suggesting that additional, divergent HEPN domains were likely to exist that might be difficult to detect using the standard iterative search strategies alone. Hence, we resorted to a two-pronged search strategy. First, we seeded PSI-BLAST and HMM searches with all the borderline hits that shared the conserved motif with the HEPN domain and constructed an alignment of the corresponding regions of the sequences that yielded significant hits in these searches. These alignments then were used to initiate profile-profile searches with the HHpred program against a library of profiles based on Pfam, Interpro and those prepared using sequences from the PDB structural database [44]. Second, we initiated HHpred searches using profiles of known HEPN domains (i.e. the models from the HEPN clan of the Pfam database augmented by the new members recovered in our searches) against the same library of profiles as in the first approach. We then selected all query alignments that recovered a known HEPN profile as the best hit as candidate novel HEPN domains. Each of these candidates was analyzed using secondary structure prediction, with the JPRED program, examination of conserved motifs, transitive recovery of known HEPN domains in profile and HMM searches, and additional profile-profile searches to test their membership in the HEPN superfamily (see Methods for details).

The search strategy outlined above identified numerous families of domains (typically with probability of profile match in HHpred of 75-98%; Table 1 and Additional file 1) as being new versions of the HEPN domain. Strikingly, we observed that several of these newly recognized families correspond to the catalytic domains of RNases that have been previously biochemically characterized. These include the mRNA cleaving RNase LS family implicated in the defense against enterobacteriophage T4 [48,49], the tRNA anti-codon loop-cleaving RNase domains of RloC [50] and PrrC [51] also involved in the restriction of T4 [52], and the kinase-extension nuclease (KEN) domain of RNase L which is involved in specialized splicing reactions and interferon-induced antiviral response in vertebrates [53,54]. Consistent with these results, we also detected a novel version of HEPN domain in the pan-eukaryotic Las1 proteins involved in the cleavage and processing of the ITS2 linker RNA which separates the 5.8S and 25S/28S rRNAs in their common precursor [55]. The eukaryotic Swt1 proteins, which are involved in the degradation of pre-mRNA at the nuclear pore to prevent their exit to the cytoplasm [56], also displayed a previously unknown version of HEPN domain.

**Table 1:** Classification, domain architectures, gene-neighborhoods and other salient features of HEPN proteins

**Family (with any Pfam names/id)**	**Conservation of Rx4-6H**	**Salient Architecture and operons**	**Phyletic pattern, available structures and comments**
**Nucleotidyltransferase (NT-) associated HEPN families**			
HEPN-T (PF05168)	D replaces conserved H in several cases	Standalone versions and fusions to MNT; In the case of Sacsin it is part of a multi-domain protein with vertebrates showing a further fusion to an Ubiquitin-like domain and some animals showing a fusion to a Death domain. Several instances of genomic clustering with R-M system operons	Bacteria, Archaea, Eukaryotes
			pdb: 1wwp, 2hsb, 1o3u.
			Proteins with conserved D in place of H have a conserved H elsewhere which could contribute to activity
HEPN-T(Parep1/8)	Lacks R but H is conserved	Fused to inactive LAF-1/Vasa-like RNA helicase N-terminal ATPase domain in *Caenorhabditis.* In operon with genes encoding Parep in tandem repeats or with genes encoding proteins with MNT and REase (DUF1626)	Archaea. Has two distinct families PAE0096 and PaREP1. PDB:2q00
HEPN-T (Cpin_6617)	No	Fusions to a dyad of ferredoxin domains (gi: 381187024, *Bacteroidetes*, *Nitrospirae*)	Mostly Bacteria
HEPN-M (PF08780/DUF86-PF01934)	Mostly conserved (83%)	Occasionally fused to MNT, a previously undetected archaeal Holliday junction resolvase-like REase (Additional file 1), and nucleic acid methylase domains. In operon with a HAD phosphoesterase gene	PDB: 1ylm, 1jog-A. Bacteria, Archaea
HEPN-M (SAV_6107)	No	-	actinobacteria
Aminoglycoside_NT_C (PF07827/DUF4037)	No	Found at the C-termini of aminoglycoside nucleotidyltransferase and related proteins (gi: 15923025). Occasionally fused to TPRs (gi: 296454793)	PDB:1kny, 3jyy, 3jz0, 2pbe Bacteria
GlnD/GlnE (PF08335)/ DUF294_C (PF10335)	No	Fused to GlnD/E-like nucleotidyltransferase. Usually part of the glutamine synthetase modifying complex. DrrA is a secreted toxin in *Legionella*.	PDB:1v4a, 3l0i Bacteria
**DUF4145-like**			
DUF4145 (PF13643)	Mostly conserved (80%)	Fused to Restriction Endonuclease (REase, SF-II-Helicase); Sel1, Zinc Ribbon, TM and SH3 (Firmicutes), UvrD Helicase (endoV alpha subunit), TIR and ATPase (*Thiorhodococcus drewsii* AZ1); SIGMA-HTH; DpnII/MboI-NTD; AbiJ-NTD1.	Bacteria > Archaea^a^, dsDNA viruses;
		In operon with R-M, TerD, McrB/C and symE toxin	
c2405	Conserved H but lacks R	Fused to N-terminal AbiTii domain and in a few cases to a C-terminal Helix-hairpin-helix domain	Bacteria
MtlR	60%	Most often a part of mannitol operon with other mannitol utilization genes	gamma proteobacteria pdb:3c8g, 3brj
**Abi2/Swt1**			
Abi2/AbiF/AbiD	Yes	Abi2/AbiF/AbiD and jhp1408 families	Bacteria
		Embedded in R-M operons and also a protein with DNase domains ParB and HNH (*Victivallis*, *Fusobacterium*);	
Swt1-like	Partly conserved	**Swt1** - Dyad of HEPN domains fused to a PIN domain, with an additional fusion to WW in some; Inactive.	**Swt1** - Eukaryotes
		**Ava_2192** - HEPN fused to a novel AAA + −ATPase. The Pfam profile DUF499 overlaps with this AAA + −ATPase (See Table 2); In operon with R-M components, where the SNF2-Helicase is fused to DUF3883, which is a novel REase domain. Active.	**Ava_2192** - Bacterial with transfer to *Naegleria,* Dictyosteliida*, Daphnia* (expansion). All eukaryotes are solos.
		**Cxorf38** - Zn ribbon inserted into HEPN, DSRBD, NACHT, Ankyrin, CARD and DEATH, Active.	**Cxorf38** - Vertebrates, *Saccoglossus,*
		**PY00838** – Fusion to Aegerolysin (*Apicomplexa*, Inactive)	*Branchiostoma, Ciona, Nematostella*. The Human gene is highly expressed in B lymphoblasts and CD56+ NK cells suggesting that this group might be involved in RNA virus defense.
		**Other Fusions** to TM (STY4199), active; Phospholipase D Nuclease (SAV_2148 ), inactive; ParB (Saro_3948), mostly active; and ParB (DUF262) with HNH(DUF2081) (VNG7073 ), active; Transglutaminase, SF-I-	
		Helicase, Vsr REase and 2 wHTH (MTES_1575), active; CBS and HD (alr3009), active; RNASEIII and DSRBD (Cyanobacteria), active; STAND-ATPase, TPR, S1 (Npun_F6454, MED222_16016, Desac_1927), mostly active; SWI2/SNF2-ATPase (WQE_15321), active; Zinc Ribbon (Npun_R5629); ZnR with two TMs (Plim_2023), active.	
Ribo L-PSP-HEPN	Yes	Fused to endoRNase L-PSP(gi: 166363853) ; operon with ParB	Bacteria. Distantly related AbiF and AbiD
**Other Abi**			
AbiU2	Yes	In operon with a gene encoding protein with Sel1 repeats; R-M operons;	Bacteria
AbiV	No	-	Bacteria; Has an alternative conserved H at the same position as the first HEPN-T family; hence, could be related to that family
AbiJ	Yes	Fused to various novel N terminal domains labeled AbiJ-NTD1 to 5; Some of the solos occur in operon with R-M system	Bacteria
AbiA-CTD	Yes	Fused to Reverse Transcriptase ; in operon with R-M system	Bacteria
**MAE_28990**			
MAE_28990	Yes	In operon with a ParB nuclease and DNA methylase genes	Bacteria
MAE_18760	Yes	Fused to HEPN/RES-NTD1, HEPN/Toprim-NTD1, Schlafen and a novel beta rich domain. In operon with ParA/Soj ATPase of SIMIBI-type GTPase fold	Bacteria
**CRISPR-Cas**			
Csx1( MJ1666)	Yes	A dyad of HEPN domains fused to a Rossmann fold domain (PF09455)	Archaea > Bacteria; PDB:2i71, 4EOG
Csx1(TM1812)	Yes	HEPN fused to a Rossmann fold (PF09455), and a few other novel domains	Bacteria;
Csm6	Yes	HEPN fused to Csm6 (PF09659) and a helical domain	bacteria;
Csm6 (Cas_Cas02710)	Yes	HEPN fused to Csm6 (PF09670)	Bacteria > Archaea;
**Other families**			
Ymh (PF09509)	Yes	Solos and fusions to pMORC, AbiJ-NTD1 and AbiTii domain.	Bacteria > Archaea
		In operon with R-M	
C6orf70	Yes	Fused to TPR; WD40 (*Dictyostelium*).	Bacteria > Eukaryotes. Overlaps with DUF4209 (PF13910). This family can be traced to LECA
		Occurs in R-M related operons	
DUF2526 (PF10735)	Yes	None detected	Gammaproteobacteria
KEN (RnaseL/Ire1)	Mostly conserved (95%)	Fused to S/T/Y-Kinase, along with ankyrin repeats, CCCH in some. Also found fused to UBI (gi:125543109) and BRCT (gi: 218187285)	Eukaryotes. pdb:3lj2; solo RNase L in *Oikopleura* and an independent LSE of the same is also seen Plants (mainly monocots)
Las1	Yes	Mainly Solos. Sometimes fused to Metallo-beta-lactamase and EF-HAND (Ascomycota) and to family specific globular domains	Eukaryotes
Rnase LS	Yes	Fused to RNase H (gi: 300902643), along with Caulimovirus viroplasmin domain (gi: 222100146). In some a TATA-binding protein (TBP)-like domain replaces the RNase H fold domain. In operon with antitoxin RnlB	Bacteria
DZIP3/ hRUL138	Mostly conserved	Fusion to TPR, Zn-ribbon, RING, Ankyrin, CARD, NACHT ATPase, DEATH and LRR in various animal lineages	Eukaryotes. Mainly animal lineage: LSEs in *Nematostella*, and the oyster and *Capsaspora*
PrrC/RloC/ APECO1_4465	Yes	Fused to ABC-ATPase. Often found in R-M operon and with genes for RhuM-like or Fic/Doc-like toxins. APECO1_4465 is also found in prophages	Bacteria
ERFG_01251	Yes	Fused to ABC-ATPase and HEPN/TOPRIM-NTD1	Bacteria
ApeA/BMEI1217	Yes	In epsilonproteobacteria embedded in R-M operons	Bacteria > Archaea;
EC042_2821	Yes	Fused to wHTH, REase and ZnR domains. Occurs in R-M system operons	Bacteria overlaps with DUF3644
Integron cassette HEPN	Yes	Part of mobile integron element	PDB:3jrt Gammaproteobacteria
pEK499_p136_Ecoli like (B)	Yes	Some in operon with R-M genes, ADP-ribosyltransferase-like enzymes (ART), and Macro. Also found in operon with NamA-like RNase H fold nuclease and with the Pgl components	NamA toxin / RlfA Replication in Phage P1 has a RnaseH fold
LA2681	Yes	Fused to TPR, and in operon with TPR	Bacteria > Archaea
Cthe_2314	Yes	None detected	Bacteria
Bxe_C0808	Yes	In operon with AbiU2	Bacteria

a: The “>” sign indicates a postulated transfer from one lineage to another.

Many of the newly detected HEPN families showed additional connections to antiviral defense functions. Most notably, 6 families of domains, respectively typified by the AbiD, AbiF, AbiJ, AbiU2, AbiV and the C-terminal domain of AbiA, which are products of the eponymous abortive phage infection genes from *Lactococcus lactis,* were characterized as novel versions of the HEPN domain (Table 1). We also identified novel versions of the HEPN domain that comprised the C-terminal modules in a large group of COG1517-related proteins (including Csx1 and Csm6 subfamilies), which are encoded by genes found in a subset of the CRISPR-Cas loci (Table 1) [4,57]. These findings suggested previously unappreciated roles of HEPN domains in RNA-processing, both in defense and in cellular RNA maturation. Importantly, these observations raised the possibility that at least a subset of HEPN domains might function as RNases with diverse target specificities.

Beyond the above noted families, our analysis recovered at least 38 distinct families of domains that belong to the HEPN superfamily several of which can be further grouped together into higher order assemblages based on preferential recovery in profile or profile-profile searches (Table 1 and Additional file 1). These include functionally enigmatic families such as the MtlR family of regulatory proteins typified by the *Escherichia coli* mannitol operon regulators [58]. Other new HEPN domain families are labeled as “domains of unknown function” in the PFAM database, namely DUF3644, DUF4209, DUF2526, Ymh. Other domain families identified for the first time in this work were previously completely uncharacterized (Figure 1, Table 1). To better understand the biochemistry and biological roles of the HEPN domain we systematically analyzed the sequence features, potential active sites, structural variations and contextual connections of the HEPN superfamily proteins.

### Conserved sequence features of the HEPN domain: prediction of an RNase catalytic site

We first aligned individual families using the MUSCLE, KALIGN and PCMA programs (Figure 1, see Methods and Additional file 1) and used the resulting alignments to predict secondary structure using the JPRED program. These alignments and predictions were used to generate a comprehensive structural alignment of the HEPN domain superfamily, guided by secondary structure predictions, the results of the profile-profile searches with HHpred, and structural alignments generated by the DALIlite program. Examination of this alignment indicated that the domains are usually approximately 100–120 amino acids long which is similar to the size of the originally defined HEPN domain. However, certain families contain long inserts up to 60 amino acids in length at different points in the domain. The original analysis of the HEPN domain identified a conserved motif, Rx4H (where x is any amino acid) [42]. In the present analysis, this motif emerged as the most strongly conserved feature of the HEPN domain which is either strictly or partially conserved in almost all the families detected in this study (Figure 1). However, with the detection of the new HEPN superfamily members, the spacing between the conserved arginine and histidine in this motif was found to be more variable, with some families showing a 6 residue spacer instead of the typical 4. When the Rx4-6H motif is conserved, the residue immediately after the conserved R is typically polar (mostly N, D or H). Notably, the Rx4-6H motif is entirely or partly lost in the HEPN domains that are fused to the C-termini of nucleotidyltransferase domains (potential substrate-binding domains) and a subset of the MtlR family. Many of the HEPN families (both those that possess the Rx4-6H motif and those that lack it) contain a second conserved acidic residue, typically as part of a Ex3 [KR]motif. Beyond these elements, the rest of the domain sequence is poorly conserved between different families (Figure 1). Thus, for several of the families, which include no proteins with solved structures, the alignment outside of the conserved motifs should be viewed with caution.

**Figure 2:**
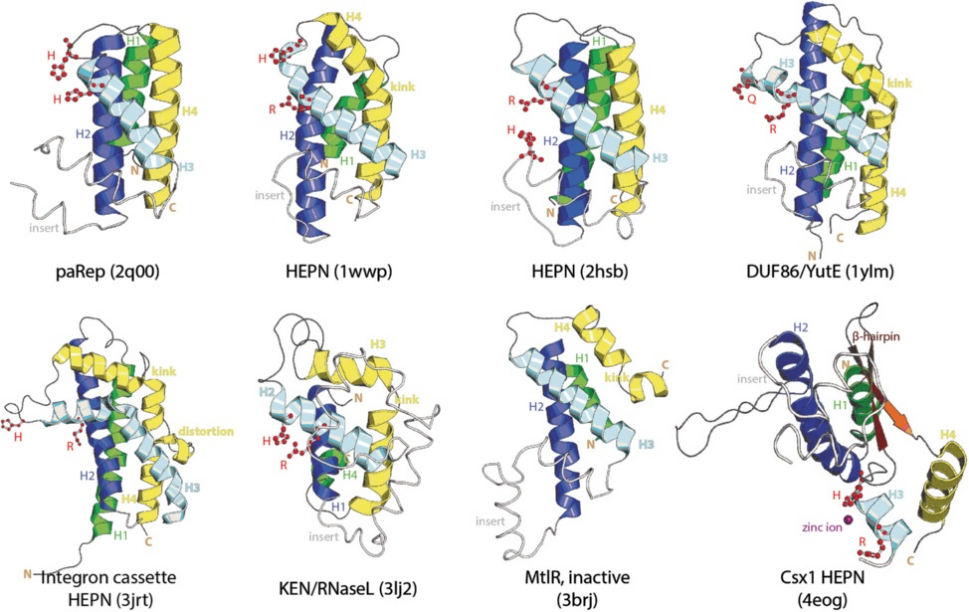
**Structural diversity of HEPN domains.** A member of each of the seven HEPN families with solved crystal structures is rendered as a cartoon; labels provide HEPN family name and PDB ID. Equivalent core helices are colored the same across all structures while labeled in the order observed from the N-terminus to the C-terminus to highlight circular permutations. In the canonical configuration, helix-1 (H1) and helix-2 (H2) from the first α-hairpin are colored green and blue, respectively and helix-3 (H3) and helix-4 (H4) from the second α-hairpin are colored cyan and yellow, respectively. The conserved insert region found between helix-2 and helix-3 in the canonical configuration is colored and labeled in light grey in each cartoon. The kink and further distortions are labeled in yellow. Conserved active site residues are rendered as ball and sticks and colored and labeled in red. Note the structural reorganization of HEPN domain in the Csx1 family. The distinctive β-hairpins of this family are colored and labeled in brown and the zinc ion found in the vicinity of the active site residues is rendered as a sphere and colored in purple.

Site-directed mutagenesis of the KEN domain of RNase L and the RNase domains of RloC and PrrC have shown that the histidine corresponding to the conserved H in the Rx4-6H motif is essential for their respective nuclease activities [53,59,60]. At least in the case of the KEN domain [53] and PrrC [60], the conserved arginine from this motif was also found to be necessary for the RNase activity. Furthermore, in the KEN domain the conserved polar residue immediately following the conserved R also appears to contribute to catalysis [53]. All these RNases appear to function as metal-independent enzymes that generate a cleavage product with a terminal 2′-3′ cyclic phosphodiester linkage. Taken together, these observations suggest that the conserved Rx4-6H motif of the HEPN domain is the primary determinant of a novel RNase active site. This active site is probably further augmented by the above-mentioned conserved acidic residue, which is typically found further upstream, and the polar residue occurring immediately after the R. Thus, the HEPN domain active site seems to resemble the colicin E3 family of metal-independent RNases in which the catalytic site encompasses a single catalytic histidine in conjunction with acidic residues [2,61]. By analogy to the colicin E3 domains, the conserved H in the HEPN domain can be predicted to induce the 2’OH to attack the phosphodiester backbone of the RNA. The conserved R could either stabilize the intermediate during the nucleophilic attack or interact with the backbone of the substrate. Although the catalytic mechanism is metal- independent, in some cases a metal ion from the vicinity of the Rx4-6H motif might stabilize the reaction intermediate further as suggested by the presence of a Zn^2+^ ion in the crystal structure of the *Pyrococcus furious* Csx1 HEPN domain (Figure 2) [62]. The identification of the conserved motif in the HEPN proteins as a potential RNase active site implies that all HEPN domains that possess this conserved motif function as RNases (Table 1). This proposition is supported by the detection of RNase activity across widely divergent HEPN domains: while both the KEN and the PrrC/RloC RNase domains are, each in their own way, distinct in sequence and/or structural features from the originally identified HEPN domains, the RNase LS and LsoA nuclease domains are typical HEPN domains (Figure 1, Table 1 and Additional file 1).

Certain families HEPN domains show variations in the conserved motif: (1) in the Parep1/8 family of the HEPN-T clade (Table 1, Figure 2) the H is present but the R from the Rx4-6H is not conserved. However, this family contains other strongly conserved basic residues elsewhere in the sequence that could have taken over the function of the R (Additional file 1). In certain families like AbiV and the HEPN-T family typified by *Archaeoglobus fulgidus* AF0298 (pdb: 2hsb) we observed that the H is typically absent in the Rx4-6H motif though the basic residue corresponding to the R is present. However, these proteins have a second strongly conserved H occurring N-terminal to the above motif. Examination of the structure reveals that this H could constitute an alternative active site similar to the classical HEPN active along the basic residue from the motif (Figure 2). Thus, it is possible that certain families of HEPN domains, which lack the canonical form of the motif, still catalyze cleavage of RNA by utilizing alternative active-site residues. A comparable use of alternative active sites for RNase activity while sharing a common fold has also been noted in other structurally unrelated folds of RNases such as the barnase-EndoU-colicin E5/D-RelE fold (the BECR fold) [2] and the RAMPs from the CRISPR-Cas systems [63]. However, HEPN domains entirely lacking any conserved charged and polar residues are likely to be catalytically inactive versions that function as nucleic acid-binding domains.

### Structural features of the HEPN domain and the remarkable structural rearrangement in the HEPN from CRISPR-Cas systems

To place the identified sequence features of the HEPN domain in a three-dimensional context, we performed a systematic comparison of all available structures of HEPN domains in the PDB database. Other than the C-terminal helical domains of nucleotidyltransferases (see SCOP database id: 81593 [64]), we retrieved 16 distinct structures of HEPN domains that come from 7 distinct families (Figure 1 and Table 1). A comparison of these structures showed that the HEPN domain adopts a four-helical up-down fold similar to the fold of the coat proteins of plant rod-shaped RNA viruses (e.g. tobacco mosaic virus) and cytochrome C (Figure 2). The core of this fold has a simple architecture comprised of two similarly structured α-hairpins that are appressed against each at an acute angle such that the N- and C-termini are spatially juxtaposed. Such an arrangement of the termini can favor circular permutations [65], which is indeed observed in the structure of the KEN domain (Figure 2), where the equivalent of helix-1 of typical HEPN domains becomes the C-terminal-most helix. However, the HEPN domain is distinguished from other domains with a comparable four-helical fold by the frequent presence of inserts between helix-2 and helix-3 which assume the form a long loop, an additional helical element or even a helical hairpin (Figure 2). The sequence of this insert is poorly conserved, causing most of the uncertainties in the sequence alignment. Additionally, in several of the HEPN domains helix-4 is either kinked (e.g. the HEPN domains encoded by genes adjacent to MNTs, PDB: 1wwp and YutE, PDB: 1ylm) or further distorted by residues in non-helical conformations (e.g. the integron-associated HEPN domain, PDB: 3jrt and the KEN RNase domain: 3lj2). The Rx4-6H motif is situated at the end of helix-3 and in the beginning of the loop connecting helix-3 to helix-4. The histidine in this motif is always exposed to the solvent and available for catalysis. The conserved acidic residue in N-terminal part of the HEPN domain (Figure 2), when present, is in helix-2, and is positioned proximal to the above motif, supporting its role in the nuclease active site of the HEPN domain. The alternative conserved histidine observed in the AbiV and AF0298-like HEPN-T proteins comes from the above-mentioned inserted between helix-2 and helix-3.

In several HEPN domains the region containing the Rx4-6H motif displays residues in non-helical conformations, resulting in distortion of the helical axis in the C-terminal portion of helix-3 (e.g. PDB: 1wwp and 3jrt; Figure 2). This distortion could indicate selection for flexibility in this region, which might be required for effective catalysis or for binding the nucleic acid substrate. A more dramatic structural distortion both in this region and elsewhere is observed in the Csx1 family which is one of the four related but distinct HEPN domains found in the type I and III CRISPR-Cas systems (Table 1) [57]. All four families are predicted to be active RNases given the strong conservation of the Rx4-6H motif but they are extremely divergent from each other. Currently, structures are available for Csx1 from *Sulfolobus solfataricus* (PDB: 2i71) and *Pyrococcus furiosus* (PDB: 4eog) [62] and the *P. furiosus* Csx1 protein has been shown to bind DNA [62]. The Csx1 structure is substantially different from the structures of all other HEPN domains although the homology of Csx1 with other HEPN domains is supported by multiple profile-profile searches (Additional file 1). Comparison of the Csx1 structure protein with the predicted secondary structures of the three other families of CRISPR-Cas-associated HEPN domains suggests the Csx1 family underwent a complex structural transformation while preserving the active site motif of the HEPN domains (Figure 2). This transformation appears to have been facilitated by multiple inserts, namely a β-hairpin immediately after the Rx4-6H motif, and another large, poorly structured insert between helix-2 and helix-3. The dramatic structural distortion of the HEPN domain in the Csx1 family is reminiscent of massive structural rearrangements that apparently occurred in the evolution of the pseudo-KH and LIM domains while preserving key interaction interfaces [64,66,67].

### Inference of biological roles of HEPN domain proteins from contextual information

**Figure 3:**
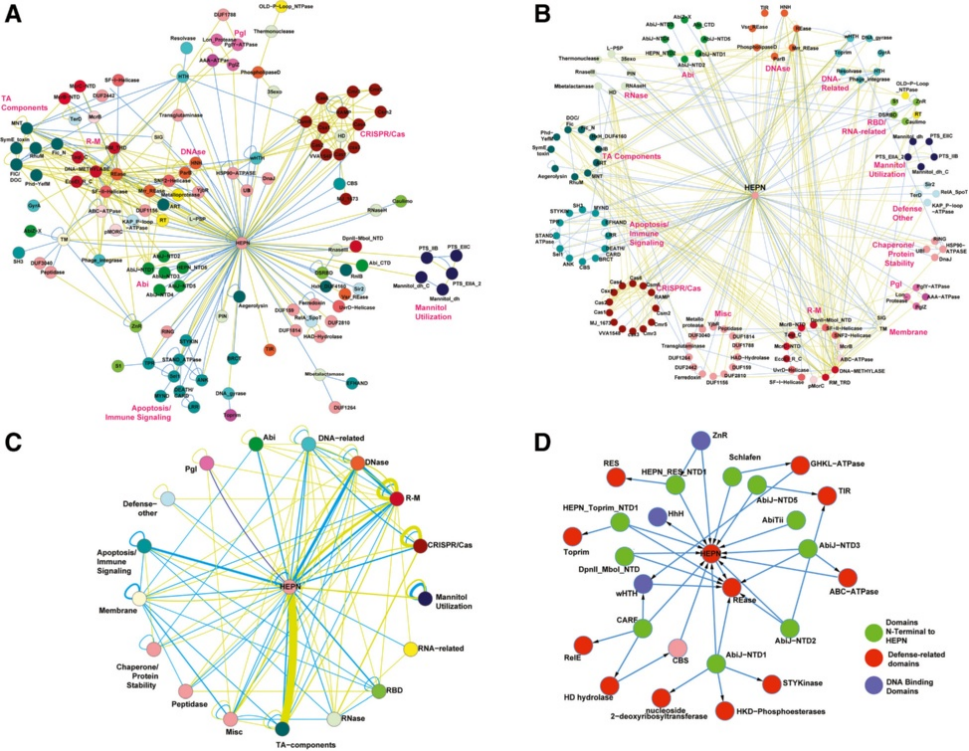
**A domain architecture and gene-neighborhood network showing the manifold functional connections of the HEPN domain.** The graphs were rendered using the Cytoscape program [73]. The network is an ordered graph with the cyan edges representing the connection between adjacent domains combined in the same polypeptides and the gold edges representing the context in the gene neighborhood. **(A)** The “force-directed” network was derived using the spring-embedded layout utilizing the Kamada–Kawai algorithm, which works well for graphs with 50–100 nodes [74]. The natural clustering of the functional categories emerging from this algorithm is indicated with labels. **(B)** The nodes of the network arranged by function. **(C)** Condensed network, where the domain belonging to a given functional category has been collapsed into that category name. **(D)** A domain architecture graph of HEPN and the various N-terminal domains which co-occur with other defense-related domains, showing the interchangeability of HEPN and the defense-related domains.

Despite identification of HEPN domains in some well-studied protein families, the biological functions of the majority of the HEPN domains remain obscure. Hence, we employed several sources of contextual information [68-71] in an attempt to infer the functions of the uncharacterized HEPN proteins and better understand those for which some functional information was available. First, we systematically collected HEPN domain-containing proteins from the non-redundant database (See Additional file 1) and determined their phyletic patterns (i.e. the patterns of presence-absence in different taxa [72]). Next we determined the domain architectures of these proteins by searching their sequences with a library of sequence profiles derived from the PFAM database augmented with additional in-house collections of profiles for domains involved in nucleic acid metabolism, signaling, and organismal conflicts. In cases where there were conserved globular domains associated with the HEPN domain, which did not hit any previously recognized domain, sequence-profile and HMM searches were carried out to further characterize these domains (Table 2 gives a list of novel domains associated with HEPN domain that were detected in this work). Thus, we generated a comprehensive collection of domain architectures for the HEPN domains. In the case of the prokaryotic representatives, operons or conserved gene-neighborhoods were inferred using genomic information and the resulting inferences were employed to predict functional associations based on the tendency of products of genes co-occurring in operons to functionally interact [68,69]. To understand the broad functional tendencies among HEPN proteins, we represented their domain architectures and operonic contexts as networks, where the nodes are individual domains and the edges represent connections in the form of fusion within the same polypeptide or co-occurrence in operons (Figure 3). We discuss below the salient findings emerging from this analysis.

**Table 2:** Selected novel domains fused to HEPN domains

**Domain name**	**Domain size (Representative and the domain range)**	**Structural and sequence features**	**Comments**
AbiJ-NTD1	~ 140 aa; (e.g. 1 to 140 aa,	Mostly alpha helical	Fused to HEPN families: AbiJ, DUF4145 (gi: 113972064), Ymh (gi: 148556575). It is also found fused to other domains potentially involved in biological conflicts: HKD-Phosphoesterases (gi:302346766), STYKinase (gi:47459341), REase (gi: 358072046) and flavodoxin fold nucleoside deoxyribosyltransferase (gi: 397664865)
	gi: 134296193, Bcep1808_2091		
	*Burkholderia vietnamiensis G4*)		
AbiJ-NTD2	~ 100 aa; (e.g. 1 to 102 aa,	Mostly alpha helical with a conserved beta strand next to the first alpha helix	Found fused to AbiJ, and to other domains presumably involved in other domains potentially involved in biological conflicts: Mrr family REase (gi: 91784007), TIR nuclease (gi: 269963288). Many AbiJ_NTD2 sequences have been erroneously included in the DUF3644 Pfam model, a new HEPN domain described here. However, profile-profile searches do not demonstrate an independent relationship between AbiJ_NTD2 and HEPN independently
	gi: 60680647, BF1118, *Bacteroides fragilis NCTC 9343*)		
AbiJ-NTD3	~ 140 aa; (e.g. 1 to 142 aa,	Alpha + beta	Found fused to AbiJ. Fused to other domains presumably involved in defense: ABC ATPase (gi:319955098), REase domains prototyped by the Pfam model DUF2726 (gi:56476843)
	gi:187251857, Emin_1454,		
	*Elusimicrobium minutum* Pei191)		
AbiJ-NTD4	~ 160 aa; (e.g. 1 to 165 aa,	Alpha + beta	Found fused to AbiJ and heat repeats (gi: 71907952)
	gi: 182417316, CBY_0614,		
	*Clostridium butyricum 5521*)		
AbiJ-NTD5	~ 100 aa; (e.g. 1 to 115 aa,	Mostly alpha helical	Found fused to AbiJ, and to other domains presumably involved in defense: TIR nuclease (gi:296123260), some have a further N-terminal DnaG-like CxxH-CxxC Zn ribbon domain
	gi: 149930787, w0043, *Escherichia coli*)		
AbiTii	~ 180aa; (e.g. 1 to 180 aa of gi: 358446093)	Alpha + beta	Found fused to the N-terminus of the c2405 family of HEPN domains and in few cases to Ymh (gi: 372210551)
HEPN/RES-NTD1	~ 100 aa; (e.g. 1 to 95 aa,	Mostly alpha	Fused to HEPN (MAE_28990 superfamily), RES domain, a potential RNase found in various toxin
	gi:206576331, KPK_1764 *Klebsiella*	helical	systems (gi: 30248753). Also occasionally fused to an ABC ATPase and two other novel domains (Supplementary material). Some of those fused to RES have a further N-terminal Zn ribbon domain
	*pneumoniae* 342		
HEPN/Toprim-NTD1	~240 aa; (e.g. 1 to 240 aa, gi: 423201025; HMPREF1167_01188 *Aeromonas veronii*.	Alpha + beta	Fused to two distinct HEPN families: MAE_28990 and ERFG_01251 families (gi: 118587223), TOPRIM (gi: 160895002) and a Mrr-like REase domain (gi: 383455290)
DpnII/MboI-NTD	~100 aa; (e.g. 1 to 115 aa	Mostly alpha helical with a conserved beta strand next to the first alpha helix	This domain can be unified the α-helical domain found at the N-termini of the type-II REases DpnII and MboI. I It is fused to the HEPN domain prototyped by the Pfam DUF4145 model and to other domains presumably involved in defense: e.g. a novel REase (gi: 146284642)
	gi:218440340, PCC7424_3406,		
	*Cyanothece sp. PCC 7424*)		
ApeA-NTD1	~ 300 aa; (e.g. 1 to 300 aa,	Mostly beta strands	Fused to HEPN (Apea). Several conserved aromatic residues, abundant but poorly conserved
	gi: 218441941 PCC7424_5050,		
	*Cyanothece sp. PCC 7424*)		
MAE_18760-NTD1	~121 aa; (e.g. 1 to 121 of gi: 385800275)	Mostly beta strands	Found at the N-terminus of certain members of the MAE_18760 family
AAA-ATPase (Ava_2192-CTD)	~300aa; (e.g. 160 to 460aa gi:75908411, Ava_2192, *Anabaena variabilis*)	AAA-ATPase fold	The AAA-ATPase domain overlaps with Pfam DUF499. Fused to HEPN (SWT1/Abi2 family)
wHTH	~65aa ; (e.g. 2006 to 2075 and 2095 to 2161 gi: 323358023, MTES_1575, *Microbacterium testaceum*)	wHTH fold	Fused to HEPN (SWT1/Abi2 family), along with Transglutaminase and Vsr–family REase domains. Overlaps with DUF3320.
Novel Vsr-REase (MTES_1575_REase)	~180aa (e.g., 1810..1990 aa gi: 323358023, MTES_1575, *Microbacterium testaceum*)	Vsr REase Fold	Fused to HEPN (SWT1/Abi2 family), along with Transglutaminase and wHTH.
Novel REase (EC042_2821_CTD)	~180aa (eg, 240..420aa gi: 387608267, EC042_2821, *Escherichia coli*)	REase Fold	Fused to HEPN (EC042_2821) and an N-terminal wHTH in some.

#### Evolutionary conservation and lineage-specific expansions of HEPN domains helps predict novel RNA processing and defense systems in eukaryotes

**Figure 4:**
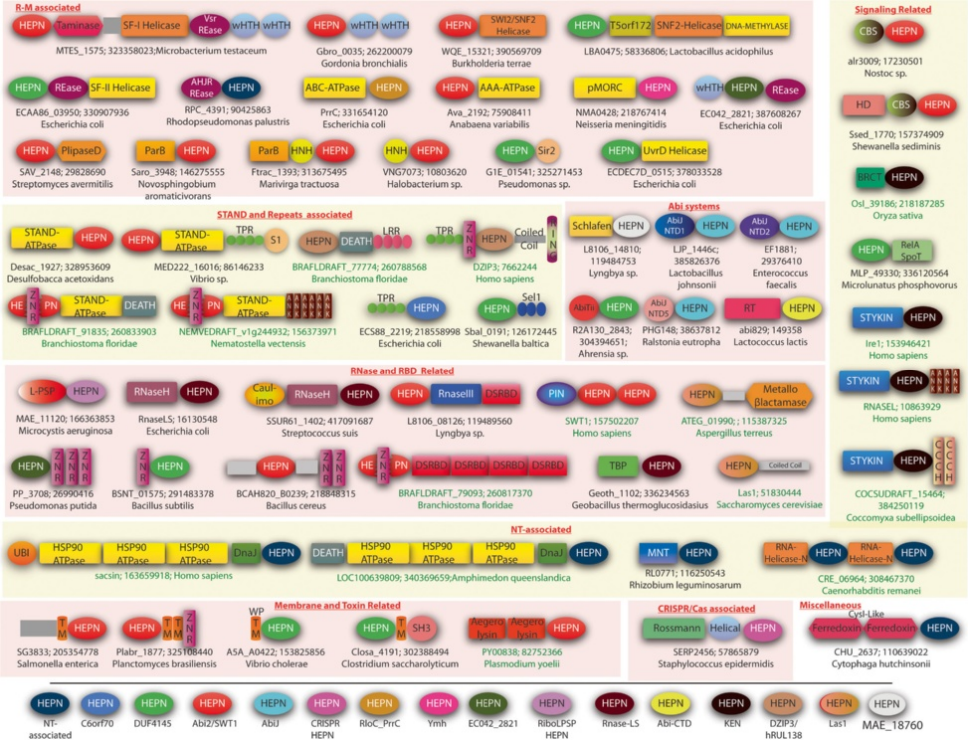
**Selected domain architectures of HEPN proteins.** The domains are not drawn to scale. Domain architectures are labeled with a representative gene name, the Genbank identifier (gi) number, and the species name separated by semicolons. The labels of eukaryotes are colored green. The generic functional categories are shown in red letters. Uncharacterized globular domains of limited phyletic spread are shown with a grey rectangle. Domain names of most domains follow the Pfam database or literature [78] (also see Additional file 1). Non-standard domain abbreviations: Ank – Ankyrin; CARF- CRISPR/Cas-associated Rossmann fold domain; PlipaseD – Phospholipase D; Taminase – Transglutaminase; TM – transmembrane helix; Helical – Helical domain.

In eukaryotes the distribution of HEPN domains shows two distinct patterns. One group of HEPN families is strongly conserved across all major eukaryotic lineages implying that they were present in the last eukaryotic common ancestor (LECA). This group includes the KEN domains found at the C-termini of serine/threonine kinase domains in Ire1-like proteins, Las1, and the family prototyped by the human protein C6orf70 (DUF4209). The KEN domain is a RNase that is involved in the degradation of rRNAs, mRNAs associated with the endoplasmic reticulum (ER) membrane, and spliceosome-independent splicing as part of the cellular response to the accumulation of unfolded proteins in the ER [53,75,76]. Thus, the emergence of the KEN domain appears to have been linked to the origin of the eukaryotic endomembrane system. The C6orf70 family, which we predict to be a catalytically active HEPN domain protein (Figure 1), similar to the Ire1-like proteins, contains a single transmembrane (TM) region and is predicted to localize to the ER membrane (Figure 4). Thus, we predict that, similar to Ire1, these proteins also function in the degradation of RNA at the ER membrane, perhaps as part of the misfolded protein response or similar stress-related regulatory processes. The identification of a HEPN domain in Las1 helps clarify key steps in the remarkably complex, eukaryote-specific processing of the ITS2 linker between the 5.8S and 25S/28S rRNAs in their common precursor [55,77]. Las1 copurifies with several exoRNases, and cooperates with the exosome and other exoRNases in processing the ITS2 linker to release the mature rRNAs [55]. However, the identity of the endonuclease required for initiating this processing event remains unknown. Based on the presence of intact catalytic residues in the HEPN domain of Las1, we predict that this protein functions as the endoRNase that makes the two initial breaks in this processing event.

The Swt1 endonuclease family, although not confidently traceable to the LECA, is inferred as being present in the common ancestor of animals, plants and fungi (Table 1). This version of the HEPN domain is fused to an N-terminal PIN endoRNase domain (Figure 4) and might be catalytically inactive due to loss of the conserved motif. Hence, we infer that this HEPN domain might have a role in binding and sensing unspliced pre-mRNAs that are specifically targeted by the Swt1 nuclease at the nuclear envelope [56].

The KEN and Las1 families, although traceable to the LECA, do not show specific relationships with any prokaryotic HEPN domains. Conceivably, these quintessentially eukaryotic variants of the HEPN domain originated by rapid sequence evolution from either a bacterial or an archaeal HEPN ancestor at the stem phase of eukaryote evolution antedating the LECA. In contrast, both the C6orf70 and Swt1 families include catalytically active bacterial versions (Table 1; see below), pointing to their origin via lateral transfer at different points in eukaryote evolution.

The other prominent trend in eukaryotes is the independent lineage-specific expansion (LSE [79]) of several families of HEPN domains. The KEN domain, separately from the N-terminal kinase domain found in Ire1-like proteins, shows independent LSEs in the tunicate *Oikopleura dioica* and several monocot plants, such as rice. Certain eukaryotes, such as the dictyostelid slime molds, the heterolobosean amoeboflagellate *Naegleria* and the crustacean *Daphnia*, possess a distinct, catalytically active HEPN domain of the Swt1 family, which is more closely related to the bacterial versions than the conserved version in Swt1 (Additional file 1). In *Daphnia* this version underwent a massive LSE with 46 distinct copies in the genome. A representative of the paREP1/8 family that appears to be of ultimate crenarchaeal origin was acquired by the nematodes of the genus *Caenorhadbditis,* where it underwent independent LSE in *C. elegans* and *C. remanei*. This pattern of multiple independent LSEs is common among eukaryotic genes with immunity- or defense-related functions: the multiplicity of diverged paralogs provides the means for maximizing the diversity of recognized targets [79-81]. Thus, it appears likely that these LSEs of HEPN domains play specific roles in defense responses. The expanded KEN and Swt1 family HEPN domains are inferred to be catalytically active and can be predicted to function as defensive endoRNases that might be directed against different viral RNAs. Several of the versions from the LSEs in the *Caenorhadbditis* species might not be active. Hence, we propose that these proteins function as receptors for targeted RNAs rather than as active RNases. Some of the nematode HEPN domains are fused to an inactive ATPase domain closely related to the N-terminal ATPase domain of the LAF-1/Vasa-like RNA helicases (Figure 4). Given that these helicases are components of the P granules of germline precursor cells, which contain RNA-interacting components that silence pseudogenes and transposons [82,83], it is possible that these HEPN proteins form a line of defense for the germline genome against selfish elements.

#### Gene-neighborhoods and protein domain architectures suggest that HEPN domains function in multi-pronged defense jointly with prokaryotic restriction-modification systems

The identification of the nuclease domains of PrrC and RloC as HEPN domains is of considerable interest because these nucleases are deployed as part of a multi-pronged defense strategy against the enterobacteriophage T4 (and most likely against other, related phages). Although PrrC and RloC are both anticodon nucleases (ACNases), which target tRNA^Lys^ of the host cell to inhibit translation during the T4 infection, each of these endoRNases has distinct biochemistry. While PrrC merely cleaves the anticodon loop, RloC excises the wobble nucleotide of tRNA^Lys^[50,59], thereby preempting the RNA-ligase-dependent phage counter-strategy. These endoRNases are part of fine-tuned defense systems that are regulated via interactions with domains in the same polypeptide and/or other proteins encoded in the same operons and whose potentially self-harming activities are deployed only at opportune moments during phage infection [52]. In PrrC and RloC the C-terminal HEPN domain is combined with N-terminal SbcC/Rad50-like ABC NTPase domains [84] (Figure 4) which regulate the activity of the nuclease domain in a manner dependent on NTP hydrolysis (in both) or sensing nucleotides (dTTP in the case of PrrC). Furthermore, PrrC is embedded in a gene-neighborhood that also encodes the three subunits (hsdMSR) of a type Ic R-M system, PrrI. This R-M system, which interacts with PrrC to keep it in a catalytically inactive state, functions as the first line of defense against the phage [51]. However, when T4 inactivates the PrrI R-M system by deploying the Stp anti-restriction peptide that is conserved in T4-like phages, or when the levels of dTTP or unmodified DNA increase, PrrC is relieved of its negative regulation [52] and steps in as a second-line of defense against the virus by inactivating tRNA^Lys^. In contrast, RloC is not linked to any R-M system but is normally kept in an inactive state by its own N-terminal ABC-ATPase domain. The HEPN nuclease domain of RloC appears to be activated when the conformation of the ABC-ATPase domain is modified in response to DNA damage from genotoxic stress induced by the virus.

**Figure 5:**
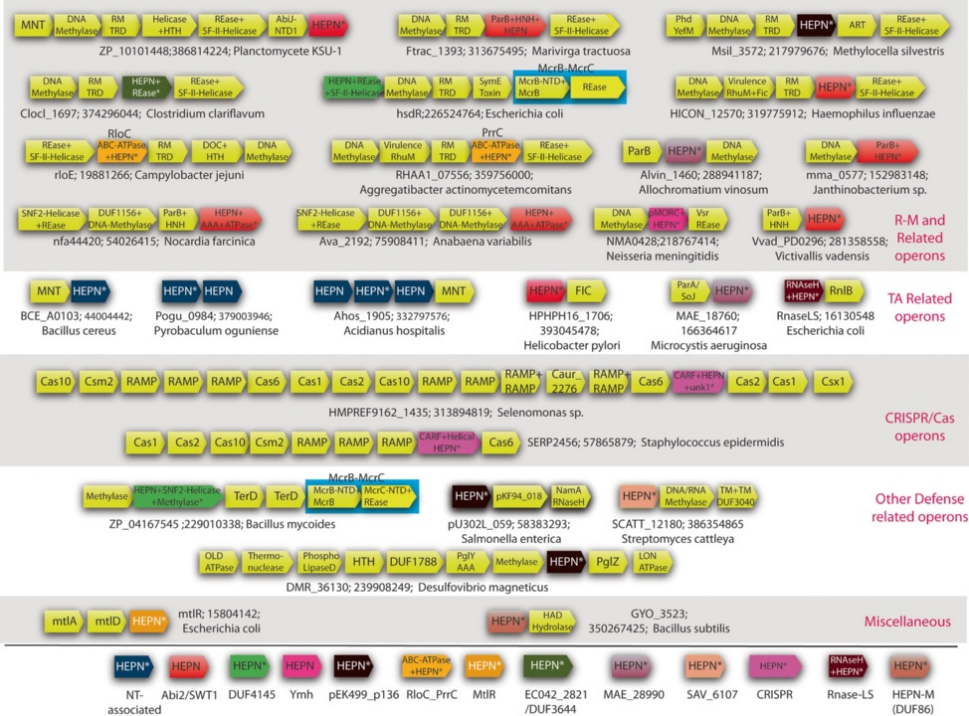
**Selected gene-neighborhoods of HEPN genes.** The gene neighborhood data for some of the genes encoding HEPN domain containing proteins is depicted using arrows. The HEPN gene is marked with an asterisk. The direction of the arrow is the direction of transcription of the gene. The gene name, Genbank identifier (gi), and the species name of the starred gene are shown next to the operon. The multi-gene modules that always co-occur are boxed. The cartoon representations of the genes are not drawn to scale. The depicted operons are typically representative of a types of operons found in a range of diverse organisms. Domain names of most domains follow the Pfam database or literature [78] (also see Additional file 1). Non standard abbreviations: RM_TRD, restriction-modification target recognition domain.

The results of these studies imply that analysis of the gene neighborhoods and domain architectures of the prokaryotic HEPN domains might help uncover multi-pronged defense strategies that evolved through the arms race between viruses and their hosts [4]. Our current analysis showed that at least 16 distinct clades of HEPN domain proteins are encoded by genes that are linked to a diverse array of R-M systems through conserved gene neighborhoods (Table 1 and Figure 5). These associations are primarily represented in bacteria where they comprise one of the most common genomic contexts of HEPN genes (Figure 3). By analogy to the PrrC-PrrI linkage, we propose that these associations between R-M systems and HEPN domains represent different multi-pronged defense strategies. A subset of RloC-like ABC-HEPN proteins are encoded within mobile gene-neighborhoods that in addition to genes for R-M components, also encode a toxin of the DOC superfamily [35] (Figure 5). The DOC domains function by NMPylating serines and threonines in target proteins and are contained in a broad variety of toxins including TA systems, polymorphic toxins and secreted effectors of pathogens [2,85]. These genomic associations suggest that the respective defense systems exercise a three-level defense strategy which targets invading DNA via the R-M system, RNA via the HEPN protein, probably by inhibition of translation, and proteins via the DOC toxin. In a similar vein, we found that some PrrC-like proteins are encoded by genomic loci that combine genes for R-M system components and those for RhuM-like proteins, which were previously observed in pathogenicity islands of *Salmonella*[86]. In these gene neighborhoods the RhuM-like protein occupies a position similar to that of the DOC toxin in the neighborhoods discussed above, and indeed the RhuM-like domain is often fused to the DOC domain. Based on this association, we propose that RhuM is also a toxin domain that might function via protein modification as part of a multilevel defense program, jointly with the PrrC-like and RM proteins. We also found that several HEPN domains of the Ymh (Pfam: PF09509) family are fused to the C-termini of ATPases of the GHKL superfamily, known as paraMORCs [87], in proteins encoded by genes embedded in R-M system gene neighborhoods. The paraMORC domains, although unrelated to SbcC/Rad50 ABC-ATPases, appear to function analogous to the latter in both R-M and other contexts [88,89]. Hence, we propose that these Ymh proteins represent an independent emergence of a domain architecture that is functionally analogous to PrrC and RloC.

Several families of HEPN domains display independent fusions to one or more of four distinct families of endoDNase domains found in R-M systems, namely domains of the REase fold (on multiple independent occasions), HNH/EndoVII fold, ParB-like fold and HKD/phospholipase D fold (Table 1 and Figures 4 and 5). In addition, we identified multiple, independent fusions of the HEPN domain with SWI2/SNF2 helicases, EcoEI-like superfamily(SF)-II helicases and SF-I helicases, which are the helicase subunits found in several distinct R-M systems (Figure 4). In one such group of giant proteins (e.g. gi: 380301041 from *Brachybacterium squillarum*), in addition to a fusion to the HEPN domain, the SF-I helicase is also fused to a transglutaminase-like peptidase, a REase fold DNase of the very short patch repair (Vsr) family and winged helix-turn-helix (wHTH) domains (Figure 4) [90-92]. In another class of R-M systems, a HEPN domain of the Abi2/SWT1 family is fused to a distinct version of the AAA + ATPase domain (currently labeled DUF499 in the Pfam database, Figure 4). Given that these RM systems typically possess a distinct helicase subunit (Figure 5), we propose that the AAA + domain fused to HEPN functions as an accessory subunit required for DNA-looping, analogous to the AAA + protein GTPase McrB in the McrBC system [93]. The above observations indicate that HEPN domains, associated with R-M systems, fuse only to restriction endonucleases, helicases and other ATPase subunits but not to the methylases. These multiple, convergent fusions imply strong selection for functional linking of the HEPN domains with DNases that cleave the target DNA and other enzymes that facilitate cleavage but not the DNA-modifying enzymes. Thus the functional analogy with PrrC is likely to extend to the HEPN domains that are associated with R-M systems. Specifically, the RNase activity of these HEPN domains is reversibly inhibited by the associated R-M system subunits but is released from this block when the R-M system is neutralized by a virus counter-strategy or in response to a genotoxic stress signal indicating that the defensive capacity of the R-M system is overwhelmed [4]. The above mentioned systems comprised of giant proteins containing HEPN, transglutaminase, SF-I helicase, Vsr DNase and wHTH domains entirely lack associated genes for DNA-modification subunits. Hence these proteins are likely to function independently of any modification, probably by directly recognizing invading DNA via their C-terminal wHTH domains. As in the case of the regular R-M systems, here too the RNase activity of the N-terminal HEPN domain is probably deployed for suicidal action if the associated DNase activity fails against the invading DNA. The fusion to the transglutaminase domain suggests that a further line of defense might involve protein cleavage catalyzed by this domain.

#### HEPN domains in bacterial RNA-based defense systems

In contrast to PrrC and RloC, the HEPN domain proteins RNase LS and LsoA, which also constitute distinct anti-phage T4 defense systems, are indiscriminate mRNases that cleave both free and ribosome-associated transcripts [49,94]. Although these endoRNases can degrade host mRNA, they appear to be primarily directed against viral mRNAs. Both RNase LS (RnlA) and LsoA are typically kept in an inactive state via physical interaction with the unstable products of the respective upstream genes, the RnlB proteins [48,49]. However, when phage T4 inhibits the production of host proteins, the RnlB proteins are removed through degradation, unleashing the RNase activity of the HEPN domain [48]. In this regard, the RnlAB system resembles Type-II TA systems some of which are deployed as defense mechanisms against phages such as P1 [48,95]. Thus, RnlAB appears to be a defense system that primarily functions at the RNA-level rather than in conjunction with any DNA-level restriction system. In most cases, the RNase LS family HEPN domain is fused to an N-terminal caulimovirus-like RNase H fold domain (e.g. gi: 335428883 from *Haloplasma contractile;* Figure 4), which in the *E. coli* RNase LS and LsoA is interrupted by a stop codon, leaving HEPN as the only active nuclease domain. The presence of this RNase H module (in particular its N-terminal caulimovirus viroplasmin domain; Figure 4) suggests that these RNase LS family proteins specifically target RNA in DNA-RNA duplexes, perhaps priming intermediates of viral replication or transcription initiation sites. Other RNase LS family HEPN domains are fused to an N-terminal TBP (TATA Binding Protein)-like domain (e.g. gi: 336234563 from *Geobacillus thermoglucosidasius*; Figure 4), similar to that fused to an RNase III-like domain in RNase HIII [96]. Given that in RNase HIII this TBP-like domain is involved in binding DNA-RNA hybrids [97], this fusion is additional evidence that a subset of the RNase LS family HEPN domains indeed target RNA in DNA-RNA duplexes.

In addition to the RNase LS family, we identified several other fusions between (predicted) catalytically active HEPN domains and other active RNase domains resulting in “two-headed RNases”. A case in point is the fusion of HEPN with a C-terminal RNase III and a dsRBD domain (e.g. gi: 119489560 from the cyanobacterium *Lyngbya*) (Figure 4). Given the specificity of RNase III and dsRBD toward RNA-RNA duplexes (e.g. in Dicer, a key component of the eukaryotic RNAi system) [98], it appears likely that these bacterial proteins cleave dsRNA targets, with multiple cleavages catalyzed by the HEPN and RNase III domains. Similarly, a distinct family of HEPN domains (e.g. gi: 389884779 *Microcystis aeruginosa*; Figure 4), which is distantly related to AbiF and AbiD (see below), shows fusions to the endoRNase L-PSP domain that is known to cleave mRNAs [99]. Hence, these HEPN proteins might also target mRNAs analogously to the members of the RNase LS family.

In addition to the fusions within a single multidomain protein, we identified three groups of HEPN proteins encoded in gene-neighborhoods that also contain a gene coding for an uncharacterized conserved protein (Table 1 and Figure 5). Sequence profile searches showed that this uncharacterized protein contained a conserved domain that it is also present in the *Photorhabdus luminescens* nematicidal toxin NamA [100]; accordingly, we named it the NamA domain. Profile-profile comparisons using the HHpred program indicated that the NamA domain contains a novel version of RNase H fold with two large inserts within the conserved core of the fold (HHpred probability 82%; Additional file 1). Nevertheless, the NamA domains retain all the key active site residues that are required for the ribonuclease activity of RNase H. Thus, these proteins are likely to be RNA-cleaving toxins. The NamA genes also co-localize, either with or without HEPN genes, with a gene coding for a KorC-like DNA-binding HTH domains, which might again point to an activity towards DNA-RNA hybrids. The NamA-HEPN gene-neighborhoods could represent yet another example of HEPN domains functioning in conjunction with other RNases. This preponderance of functional associations between multiple RNases might be indicative of a strategy of multiple cuts (as observed in the case of RloC) employed by prokaryotes to circumvent phage RNA ligase-dependent repair systems that can easily restore RNAs with single endonucleolytic breaks [51]. Furthermore, such combinations of RNases could be involved in cleavage of RNAs with complex secondary structures.

#### Major role for HEPN domains in abortive infection systems

The abortive infection (Abi) systems, of which over 22 distinct versions were first characterized in *Lactococcus lactis* (labeled AbiA to AbiV), represent anti-bacteriophage strategies that limit the spread of infection [101]. Homologous Abi-like systems are found across a wide range of bacterial lineages [102]. They appear to act both by directly targeting phage components and by causing suicide of the infected host before the release of progeny virions [101,103]. Thus, the Abi systems seem to implement a multi-layer defense strategy that is generally analogous to that of the HEPN-RM system combinations. Here we show that the primary components of 6 Abi systems define distinct groups of HEPN domains, namely the AbiA-C-terminal domain (AbiA-CTD), AbiD (including AbiD1), AbiF, AbiJ, AbiU2 and AbiV families (Table 1). Furthermore, though the originally identified *Lactococcus lactis* AbiTii protein lacks a HEPN domain, its homologs from several bacteria are found fused to two distinct C-terminal HEPN domains namely of Ymh (gi: 372210551) and the c2405 (gi: 358446093) families. The mode of action of these Abi proteins has remained largely enigmatic to date. The detection of HEPN domains suggests a unified mechanism for their action, based on the predicted RNase activity. For example, AbiD1 has been shown to be toxic to the host cell but also to interfere with the activity of the RuvC-like Holiday junction resolvase of phage bIL66 [92,101]. Furthermore, *L. lactis* AbiD1 induces cell death at suboptimal temperatures and is also toxic in heterologous systems such as *E. coli*[101]. Based on the identification of a HEPN domain in AbiD1, we propose that the wide-range toxicity of this protein is a consequence of its RNase activity. The AbiA and AbiK proteins abrogate the maturation of phage P335, primarily by inhibiting the phage-encoded Erf/Rad52-like single-strand annealing proteins via untemplated synthesis of a DNA molecule that is covalently linked to the reverse transcriptase domain [104,105]. Although the mechanisms and the targets are completely different, the activity of these proteins is comparable to that of AbiD1, in that both inhibit phage recombination. The detection of a C-terminal HEPN domain in the AbiA proteins suggests that it might also promote cell suicide mediated by the RNase activity of HEPN. AbiF causes delayed DNA replication of phage 936, possibly by interfering with replication initiation [106]. Taken together, all these observations suggest a general two-pronged mode of action for the Abi systems: i) diverse interactions with bacteriophage components resulting in inhibition of phage reproduction and ii) host cell suicide through RNA degradation mediated by the HEPN domains.

The recognition of this general mode of action for the HEPN-containing Abi systems also leads to a hypothesis on the functions of 5 novel protein domains that we detected at the N-termini of different groups of proteins of the AbiJ family and the conserved domain in AbiTii (Table 2, Figures 3 and 4). We named the former domains, which could not be unified with any previously known domains, AbiJ-NTD1-5 (after N-Terminal Domain; Additional file 1). All these domains are predicted to adopt α + β folds, with AbiJ-NTD1, AbiJ-NTD3 and AbiJ-NTD4 displaying similar predicted secondary structures (Additional file 1). Likewise, the AbiTii domain is a novel α + β globular domain with a highly conserved glutamate (Table 2, Additional file 1). We found that, in addition to being fused to the AbiJ family of HEPN domains, the AbiJ-NTD domains also occur in other proteins at the N-termini of several enzymatic domains, thereby presenting an architectural analogy to the AbiJ family. These fusions include other HEPN domains (Ymh family with AbiJ-NTD1 and AbiJ-NTD2; DUF4145 family with AbiJ-NTD1), MRR-type REase fold domains (with AbiJ-NTD1 and AbiJ-NTD2), another REase fold DNase (DUF2726 in Pfam with AbiJ-NTD3), HKD/phospholipase D fold nucleases (with AbiJ-NTD1 and AbiJ-NTD2), TIR domains, some of which might possess nuclease activity [87] (with AbiJ-NTD2, AbiJ-NTD3 and AbiJ-NTD5), protein kinases (with AbiJ-NTD1 and AbiJ-NTD3), nucleoside 2-deoxyribosyltransferases (with AbiJ-NTD1) and a distinct group of ABC-ATPase domains (with AbiJ-NTD3). Although members of the MAE_18760 family of HEPN domains (Table 1) have not been recovered among currently known Abi systems, they display architectures similar to those of Abi proteins, with fusions to three distinct NTD domains. One of these is the previously described Schlafen domain which is also found fused to other domains implicated in intra-genomic conflicts and has an important anti-viral role in metazoans [107,108] (AMB and LA unpublished observations). The second is the novel HEPN/Toprim-NTD1 domain (Table 2 and Additional file 1) that, in addition to the aforementioned HEPN domain fusion, is also fused to another family of HEPN domains (ERFG_01251; Table 1), a TOPRIM domain nuclease that is found in several defense related-contexts [109,110] (AMB, VA and LA unpublished observations), and a REase fold DNase domain of Mrr family. A third NTD (Table 2, Additional file 1), in addition the HEPN domain, also occurs fused to RES, which a toxin domain found in several type-II TA and polymorphic toxin systems, and is predicted to function as an RNase [2].

Thus, there is a strong tendency toward multiple, independent fusions of these NTDs with both RNases and DNases, as well as other catalytic domains such as protein kinases that might also function as toxins (Figure 3). Although the nucleoside 2-deoxyribosyltransferase domain is not a nuclease, it cleaves the glycosidic bond between base and deoxyribose [111]; hence this enzyme is likely to act on DNA in a manner similar to the effect of Ricin-like toxins on RNA [112]. Generally, we propose that the Abi-NTDs interact with specific targets, namely viral proteins or nucleic acids, and interfere with their functions. The C-terminal enzymatic domains of these proteins are likely to be deployed as toxins that could cause cell suicide. The NTDs also might function as antitoxins that inhibit the enzymatic activity of the C-terminal toxin domain under normal conditions, and this inhibition is relieved when the NTDs interact with viral components. Notably, AbiJ proteins lacking the NTDs more frequently occur in RM operons suggesting that they are strongly functionally coupled with RM systems as discussed above.

#### HEPN domains in CRISPR-Cas and other antivirus defense systems

Comparable to the Abi systems, HEPN domain proteins are also major players in Type I and Type III CRISPR-Cas adaptive immunity systems in archaea and bacteria [57]. There are 4 distinct families of HEPN proteins associated with CRISPR-Cas that all show a conserved domain architectural core comprised of a distinct N-terminal Rossmann fold domain, (CRISPR-Cas associated Rossmann fold or CARF), and a C-terminal HEPN domain (Figure 4). In several cases the CARF domains are fused to a different RNase (e.g. RelE, HD or PIN superfamily) or DNase (RecB-like REase fold nuclease) domains instead of the HEPN domain at their C-termini [4] (KSM, VA, A. Burroughs, EVK, LA, unpublished observations)). These nuclease-containing CARF domain proteins do not appear to be involved in spacer acquisition or spacer-sequence-dependent restriction of foreign nucleic acids in the CRISPR-Cas systems. Furthermore, CARF-nuclease proteins are also encoded by standalone genes and in certain cases by other potential anti-phage systems (e.g. a TerY-dependent system), independent of the CRISPR-Cas systems [113]. These parallel domain architectures clearly resemble those of the three AbiJ-NTD domains discussed in the previous section. Hence we propose that the CARF-HEPN proteins function analogously so that the CARF domain is a specific sensor for an invasive component (DNA or RNA) or an infection-induced metabolite, most likely a nucleotide derivative, whereas the HEPN domain acts as a suicidal RNase. Again, it appears likely that in the absence of the infection signal, CARF keeps the toxin activity of the HEPN domain in check. Thus, the CARF-HEPN proteins most likely function as an accessory to the CRISPR-Cas systems, being the final line of defense when the CRISPR-Cas immunity is overwhelmed.

Beyond the PrrC-like and RloC-like families of HEPN proteins, we detected several additional fusions of ABC-ATPases with C-terminal HEPN domains, e.g. those prototyped by the APECO1_4465 (gi: 117623091) protein from avian pathogenic *E. coli* (Table 1, Additional file 1). This group of HEPN domains is frequently found in mobile genomic islands composed of integrated prophages in several distinct bacteria (Additional file 1). A similar localization was observed for a small subset of the HEPN domains of the RloC family (e.g. gi: 209885297). Previously, prophage-encoded enzymes have been found to be an important source of anti-phage defensive mechanisms (as an extension of the mechanism to preempt super-infection) [114]. Conceivably, the prophage-encoded ABC-HEPN proteins play a comparable role in preventing infection by other phages, probably independent of R-M systems. Another group of ABC-HEPN proteins is typified by ERFG_01251 (gi: 422781011) which couples an N-terminal ABC domain with a classical HEPN domain that is more closely related to the HEPN domains associated with MNTs rather than the versions in PrrC and RloC. Sporadically, these proteins are encoded by genes embedded within CRISPR-Cas gene neighborhoods (e.g. in *Neisseria* and *Kingella*). These ABC-HEPN proteins might perform roles similar to those proposed for the CARF-HEPN proteins.

This general principle appears to be compatible with the detection of sporadic linkages between genes encoding HEPN domain proteins and some other dedicated phage resistance systems. For example, in a few instances, members of the pEK499_p136 and RloC families of HEPN domains are embedded within a large predicted operon along with genes encoding the core components of the phage growth limitation (Pgl) system that was first characterized as a defense system against lysogenic phages (e.g. phiC31) in *Streptomyces coelicolor* (Figure 5). The Pgl system appears to function by “reverse restriction-modification”: here the DNA of progeny virions produced by an infected cell is methylated by the Pgl system methylases and restricted upon reinfection by its DNase components [115]. In the Pgl operons the gene for the HEPN protein is combined with genes for the core Pgl system components, namely the phosphatase PglZ, the AAA + ATPase PglY and DNA methylase PglX and several other genes which might encode a thermonuclease-like RNase, an OLD family ABC ATPase and a Lon-type AAA + ATPase (Figure 5) [102,116] (KSM, AMB, LA, EVK, unpublished). Given the delayed action of the Pgl system, it provides immunity only after the death of the initially infected cell. Accordingly, the Pgl system is likely to spring into action in advanced stages of infection after those defense mechanisms that could potentially save the cell have failed. Hence the sporadic couplings with HEPN domain and the thermonuclease could induce cell suicide but additionally or alternatively might cleave phage RNAs to limit the phage burst size. Comparable roles can be proposed for the HEPN genes (Pfam DUF4145) that are, on a few occasions, coupled with the TerD-dependent anti-phage system that also includes a McrBC-like RM system [113].

#### HEPN proteins in MNT-associated toxin-antitoxin systems, other mobile elements and regulatory systems

The organization of genetic elements which encode the originally identified HEPN domains and MNTs clearly resembles Type II TAs suggesting that these elements are novel TA systems [10] although their mode of action remained obscure. It has been shown that the DrrA effector of *Legionella pneumophila*, that is secreted through a type-IV secretion system and contains a nucleotidyltransferase-HEPN fusion, functions as a toxin that targets eukaryotic host cells [117]. The nucleotidyltransferase activity of this protein adenylates a specific tyrosine in the host Rab1b GTPase and is essential for the toxicity of this protein [118], suggesting that the MNT is the toxic moiety. However, a recent genome-scale assay for bacterial toxins implicated the HEPN domain [43]. In several Type-II TA gene dyads the antitoxin gene occupies the 5’ position upstream of the toxin gene [2,7,10,35]. This operon organization ensures that the antitoxin is produced first and is available to inactivate the toxin as soon as the latter is synthesized. In the MNT-HEPN gene dyads, the MNT almost always occupies the 5’ position. Taken together with the predicted RNase activity of the HEPN domains, these observations strongly suggest that HEPN is the toxin and the MNT is the antitoxin in these distinct TA systems. The antitoxin activity of MNT might involve nucleotidylation of the HEPN toxin, possibly at a conserved tyrosine that is present in the C-terminal region of most HEPN domains associated with MNTs (Figure 1). Given that toxins and antitoxins of Type II TA systems typically strongly interact with each other, it is not surprising that the MNT and HEPN domains tightly interact to form a complex [119]. This interaction appears to have been exapted to utilize the HEPN domain as a substrate-binding or regulatory domain for the MNTs. Indeed, the HEPN domains that are fused to MNT domains within a multidomain protein typically lack the predicted RNase catalytic residues and accordingly are most likely inactive. Thus, “domestication” of former TA systems appears to have given rise to protein-modifying regulatory enzymes such as the nucleotidyltransferases, which regulate glutamine synthetase [120], a potential adenylyl cyclase and several enzymes such as kanamycin nucleotidyltransferase which are used as defense against antibiotics [42]. Under this scenario, the protein-modifying activity of the MNT domain was secondarily recruited as a toxin directed against eukaryotic proteins in the case of DrrA [117].

We also uncovered a similar but less common gene dyad that combines a HEPN gene of the MAE_18760 family (Table 1, Figure 5) with a gene coding for a ParA/Soj-like ATPase [121]. Given that the ATPase gene occupies a position equivalent to that of the MNT in the MNT-HEPN modules, we postulate that its product is likely to be the antitoxin whereas the HEPN protein is the RNase toxin of these novel TA systems. The antitoxin activity of the ParA/Soj-like ATPase could either involve a nucleotide-dependent conformational change in the HEPN protein or direct phosphorylation, which is consistent with the kinase activity observed in some members of this family [121,122].

A further wrinkle regarding the MNT-HEPN systems relates to the mode of action of the HEPN toxins. A subset of the HEPN domains found in these systems preserve the Rx4-6H motif (in large part represented by the DUF86 family in Pfam) or have alternative histidines and and are likely to function as endoRNases, similar to toxins in various TA systems. However, no conserved, potential active site residues are found in several HEPN domains from the MNT-HEPN systems (Additional file 1 and Figure 1). Nevertheless, the genome-scale scan for toxin proteins revealed that even HEPN proteins lacking this motif are effective as toxins [43]. Unless these proteins have evolved an alternative nuclease active site (a phenomenon observed in the BECR fold of RNases that includes the RelE superfamily [2]), it is possible that these HEPN domains exert their toxic activity via a non-catalytic mode, conceivably by binding RNA and blocking translation. Such a non-catalytic, regulatory action also might be a feature of another family of HEPN domains, which we identified in this study, the MtlR family. Although some members of this family are predicted to function as active RNases (Figure 1), a large fraction is likely to be inactive on account of the loss of the conserved motif (Table 1). The gene coding for MtlR is frequently found in an operon with mannitol utilization genes, and has been shown to function as the repressor of this operon [123]. However, it has been shown that MtlR is unlikely to act as a conventional DNA-binding transcription factor and shows no detectable interaction with the promoter/operator region of the mannitol operon [124]. Hence, inactive HEPN domains of the MtlR family might function as RNA-binding proteins that repress the mannitol operon by blocking either transcription elongation or translation.

Expansion of the MNT-HEPN systems in Archaea, along with the frequent transfer of these operons to thermophilic bacteria [10], suggests these TA systems might play some role in the thermal stress adaptation. Several chromosomally encoded TA systems are important players in stress adaptation such as dormancy and stationary phase survival in various bacteria [125-127]. Therefore, the MNT-HEPN systems that are widespread in archaeal and bacterial thermophiles might perform comparable functions. One interesting possibility is that the recovery from the accumulation of unfolded proteins resulting from high temperature or low pH shock requires translational arrest that could “buy time” for the clearance of protein aggregates by chaperones and proteolytic systems. Such translational arrest could be mediated by the MNT-HEPN module when the activity of the HEPN domain is unmasked by degradation or misfolding of the MNT component. In this regard, it is of interest to note that in extremophilic crenarchaea these systems occasionally cluster with multiple MNT and HEPN genes (Figure 5). Each HEPN protein encoded in these loci might interact with a specific set of target RNAs thereby allowing a more precise regulation of the response.

This hypothesis seems to be consistent with the presence of a HEPN domain in Sacsin from animal and slime molds (*Dictyostelium* and *Polysphondylium;* Table 1). The Sacsin gene is mutated in human patients with spastic ataxia of Charlevoix-Saguenay, a degenerative disorder of the cerebellum and spinal cord [128]. Sacsin is a gigantic multidomain protein (Figure 4) that contains a N-terminal ubiquitin-like domain, three Hsp90-like ATPase modules followed by a DnaJ domain, which recruits Hsp70 [129], and a C-terminal HEPN domain. Sacsin has been shown to function as a chaperone aiding protein folding [130] but the role of its HEPN domain has been enigmatic. We hypothesize that, in addition to acting at the protein level to relieve aggregation via chaperone action, sacsin also acts at the RNA-level via the HEPN domain. The HEPN domain in Sacsin orthologs from several animals preserves the conserved motif (Figure 1); however, in organisms like humans it is lacks the conserved motif. Thus, depending on the lineage, the Sacsin HEPN domains might either act as RNases or as non-catalytic RNA-binding domains. In either case they could inhibit translation by cleaving or binding tRNA or mRNA thereby limiting the amount of unfolded protein in the cell under stress conditions. In certain animals, there are Sacsin paralogs with N-terminal DEATH domains that are major apoptosis-mediating adaptor domains (Figure 4). It is conceivable that these proteins are part of a suicidal response that is perhaps triggered by overwhelming unfolded protein stress.

We also identified HEPN domains (e.g. the integron family; PDB: 3jrt) that are associated with certain mobile elements, such as integrons, which are major vehicles in the spread of drug resistance determinants among proteobacterial pathogens [131]. The integron cassettes are known to be activated by stress conditions, thereby allowing swapping of genetic material that might be of adaptive value [132,133]. We hypothesize that the HEPN domains present in some integron cassettes contribute to the stress response by functioning as RNases that induce dormancy by probably inhibiting translation and thus enabling survival of harsh conditions. Notably, integron cassettes often encompass also other toxin RNases such as RelE and Cas2-like proteins (VA and LA, unpublished observations) that are likely to play similar roles.

#### Bacterial membrane-associated HEPN domains and stimulus-dependent RNA degradation

In the present work, we identified at least three distinct groups of HEPN domains that are combined with TM segments. The first of these belongs to the family that overlaps with the Pfam DUF4145 family (e.g. gi: 153825856 from *Vibrio cholerae*) and contains a distinctive N-terminal domain with a single TM helix with a strictly conserved WP signature (Figures 3, 4 and Additional file 1). This TM domain is also found in several bacterial proteins where it is fused to C-terminal receiver domains (e.g. gi: 158314014) of two-component signaling systems [134] in place of the HEPN domain. A distinct group of catalytically active HEPN domains of the Abi2/SWT1 family are fused to the C-terminus of a single, well-conserved TM helix, which in turn is preceded by another conserved globular all α-helical domain (e.g. gi: 16762698 from *Salmonella enterica*). In the third group the N-terminal HEPN domain is separated from the C-terminal Zn-ribbon domain by a pair of TM helices (e.g. gi: 325108440 from *Planctomyces brasiliensis*). All these HEPN domains are predicted to be the cytoplasmic globular domains of inner membrane proteins. This localization suggests that, similar to the Ire1 and C6orf70 proteins in eukaryotes, these HEPN proteins process RNAs on the inner side of the membrane. The specialized TM segments with the WP signature and the potential external domains could act as sensors for stimuli on the cell surface, and the resulting signal could affect the HEPN domain conformation and hence RNA stability. We also identified HEPN domains that are fused to CBS domains, in some cases together with additional HD phosphohydrolase domains [135] (e.g. gi: 118580987 from *Pelobacter propionicus;* Figure 4). Given that the CBS domains sense nucleotides and their derivatives [136], these proteins might respond to such ligands to regulate RNA stability. Thus, sensing of cell-surface and soluble signals resulting in RNA degradation could be a poorly appreciated signaling pathway in diverse bacteria.

#### HEPN domains in eukaryotic host-pathogen conflicts: evidence from domain architectures

Analysis of phyletic patterns suggests that, beyond RNase L, several other distinct HEPN domains might be key players in host-pathogen conflicts in eukaryotes. This possibility was also suggested by several eukaryote-specific domain architectures that we recovered as part of this study (Figure 4). For example, in the sponge *Amphimedon queenslandica*, there are multiple Sacsin-like proteins (e.g. gi: 340377463) fused to DEATH domains, the key adaptors in metazoan apoptosis and immunity [137,138]. Proteins of the CXorf38 family, one of the novel families of HEPN domains identified in this work, are fused to double-stranded RNA-binding (dsRBD) domains in vertebrates, cephalochordates and hemichordates, and in cephalochordates and cnidarians they are fused to NACHT NTPases [139] and DEATH domains (Figures 3 and 4). Furthermore, the human CXorf38 is strongly overexpressed in B lymphoblasts and CD56+ NK cells which are key player in the vertebrate immune response [140]. The DEATH domains and NACHT NTPase modules could link the action of the HEPN domain to an apoptotic and/or defensive response in which either cellular RNAs are degraded by analogy with RNase L, or else viral RNAs are targeted. The presence of the dsRBD containing versions of the CXorf38 family is suggestive of activity on dsRNA substrates which could include RNA viral replication intermediates. Some of these eukaryotic domain architectures are also reminiscent of bacterial proteins (e.g. gi: 229028907 from *Bacillus cereus*) that often combine an N-terminal HEPN domain with NTPase modules of the STAND superfamily (which includes the NACHT NTPase [141]), and in some cases C-terminal Cold-shock protein like OB-fold RNA-binding domains [142].

We also found evidence of deployment of HEPN domains as effectors directed against their eukaryotic hosts by apicomplexan parasites. A group of HEPN domains of the Swt1 family prototyped by the MAL13P1.321 protein from *Plasmodium falciparum* was found to be conserved throughout apicomplexa. These proteins combine a pair of N-terminal aegerolysin domains with C-terminal HEPN domains (Figure 4). Aegerolysin domains perforate membranes and could facilitate protein translocation across the lipid bilayer [143]. Given that in *P. falciparum* MAL13P1.321 is expressed during intraerythrocytic development [144], these proteins might be employed by apicomplexan in the host cells. The aegerolysin domains could ensure trafficking across the bounding vacuolar membrane and thus enable the interaction between the host RNAs and the HEPN domains. Given that these HEPN domains lack the conserved motif, they probably function non-catalytically by binding specific host RNAs.

## Conclusions

Multiple groups of HEPN domains associated with MNTs are represented across most major archaeal lineages including archaea with small genomes such as *Parvarchaeum acidophilus*[145]. A second group of HEPN domains, Csx1 from archaeal Type III CRISPR-Cas systems, is also conserved across most major archaeal lineages. Both these groups of HEPN domain proteins are also widely distributed among major bacterial lineages (Table 1, Additional file 1) but show a much more patchy distribution in bacteria than in archaea. Thus, both typical HEPN domains associated with MNTs and the modified versions found in the CRISPR-Cas system apparently were present in the ancestral archaeon. In contrast, several other HEPN domain families show predominantly bacterial phyletic spread (Table 1 and Additional file 1), suggesting that these clades originated in the bacterial domain. Nevertheless, as in the above cases, these HEPN domains show patchy distributions, with closely related lineages lacking orthologous HEPN domain-containing proteins that are sometimes represented in phylogenetically distant lineages (Additional file 1). As noted above, several groups of HEPN domains show a pan-eukaryotic distribution suggesting that they were present in the LECA.

However, for most groups of the HEPN domains, the phyletic patterns strongly suggest rampant lateral mobility and gene loss as major aspects of the HEPN domain evolution in all the three domains of life along with multiple incidences of LSE in eukaryotes. Such phyletic patterns are typical of genes involved in biological conflicts, consistent with the intra-genomic clustering of HEPN protein genes with other genes implicated in such conflicts [2,35,102]. Although various HEPN domains are represented in all three domains of cellular life, none of the HEPN domain families show phyletic patterns clearly indicative of their presence in the last universal common ancestor (LUCA) of cellular life forms (Table 1). Furthermore, HEPN domains might have been encoded by fast-evolving mobile elements involved in biological conflicts even in the LUCA if not at earlier stages of evolution.

The results of the present analysis provide for a unified view of the biochemistry, biological functions and evolution of HEPN domains. This synthesis reveals a common evolutionary thread passing through what were previously considered merely analogous defense mechanisms in prokaryotes and eukaryotes. The common origin and the predicted similar mechanisms of the defense-associated HEPN RNases in prokaryotes and eukaryotes imply that these parallels are not coincidental. Indeed, there is an obvious evolutionary and functional connection between the suicidal action of the eukaryotic KEN domains and a host of prokaryotic HEPN toxins. In ecological terms, the emergence and dispersal of certain superfamilies of RNases, including the HEPN domain, could have played a major role in the emergence of cell-cell cooperation via altruistic cell suicide, thereby providing one of the important molecular bases for important evolutionary phenomena such as kin and group selection [146-148]. The suicidal action of HEPN domains during anti-phage response either by themselves (e.g. in the Abi systems) or in conjunction with other defense systems, such as R-M, CRISPR-Cas or Pgl nullifies the fitness of the cell in which it acts. Hence, survival of the suicidal genes is likely only if they offer a fitness gain via the principle of included fitness [148]. Given that bacterial colonies or cultures are often clonal [149], such mechanisms could result in kin selection [147,150]. As the HEPN proteins might be encoded by mobile elements, which tend to disperse under stress conditions [151], they might favor group selection in mixed colonies or biofilms as well. Although the altruistic suicidal action results in the death of an individual cell, HEPN and functionally similar RNase genes might spread through transfer of DNA from the dead cell to related or unrelated cells. Thus, communities of unrelated cells with potential for altruistic suicidal behavior could outperform groups incapable of such behavior, with the suicide–inducing genes dispersed within the community through DNA uptake.

The findings presented here appear to offer strong support for the recent hypothesis that proposes echeloning of directed attack on viral macromolecules (innate and adaptive immunity) and suicide/dormancy-based defenses [4]. The repeated, independent genomic co-localization of various HEPN genes with diverse defense gene clusters as well as the two-pronged attack architectures of the Abi systems point to strong selection for the functional coupling of these two types of defense systems (Figures 3, 4 and 5). Based on the analysis of gene-neighborhoods and operons, we can predict that the HEPN domains typically implement suicidal or dormancy-inducing strategies (e.g. via inhibition of protein synthesis) that might help “buy time” for the more direct defense strategies or limit infection by altruistic cell death when the immunity is completely overwhelmed. The HEPN domains and functionally similar RNases might also limit the phage burst size even when all mechanisms of defense have failed to terminate the infection. Such restriction of the virus yield could be particularly useful when coupled with “delayed action” defense mechanisms, such as the Pgl system.

The present analysis of the HEPN domain also supports the recent hypothesis that prokaryotic intra- and inter-genomic conflict systems provided raw material for the emergence of new core cellular functions in eukaryotes [1]. The emergence of an intracellular membrane system in the incipient eukaryotic cell could have resulted in a strong selective pressure for mechanisms to cope with the overloading of the ER system with unfolded proteins. Different HEPN domains from TA or other defense systems could have been recruited under this selective pressure owing to their ability to limit translation via RNA degradation or sequestration, thus facilitating stabilization and further development of the eukaryotic intracellular membrane system. A similar recruitment appears to have occurred again later in eukaryotic evolution, when a HEPN domain from a prokaryotic TA system was combined with a preexisting chaperone, Sacsin. The HEPN domains, similar to several other RNase domains found in biological conflict systems [1,2,10,35], was also recruited as a core RNA processing enzyme, Las1, whose fixation might have enabled the emergence of the unique structure of the eukaryotic 5.8S-25S/28S rRNA precursor [55]. The eukaryotic Swt1 protein containing PIN and HEPN domains also might have been acquired from a bacterial defense system and recruited as an RNase that prevents unprocessed RNAs from exiting the nucleus [56]. Finally, the repeated use of HEPN domains in apoptosis and host-pathogen interactions in eukaryotes suggests that the ancestral functions of these proteins in prokaryotes were often drafted “as is” in different eukaryotic lineages.

The findings presented here are expected to instigate and guide laboratory experiments that have the potential to illuminate numerous aspects of cellular biochemistry and biology across the three domains of life.

## Methods

Iterative profile searches with the PSI-BLAST [47] and JACKHMMER [152] programs were used to retrieve homologous sequences in the protein non-redundant (NR) database at the National Center for Biotechnology Information (NCBI). For most searches a cut-off e-value of 0.01 was used to assess significance. In each iteration, the newly detected sequences that had e-values lower than the cut-off were examined for conserved motifs to detect potential homologs in the twilight zone. Similarity-based clustering was performed using the BLASTCLUST program (http://ftp.ncbi.nih.gov/blast/documents/blastclust.html) to cluster sequences at different thresholds. Multiple sequence alignments were built using the Kalign [153], MUSCLE [45] and PCMA [154] programs, followed by manual adjustments based on profile–profile alignment, secondary structure prediction and structural alignments. Consensus secondary structures were predicted using the JPred program [155]. Remote sequence similarity searches were performed using profile-profile comparisons with the HHpred program [46]. Gene neighborhoods were extracted and analyzed using a custom PERL script that operates on the Genbank genome or whole genome shotgun files. The protein sequences of all neighbors were clustered using the BLASTCLUST program (http://ftp.ncbi.nih.gov/blast/documents/blastclust.html) to identify related sequences in gene neighborhoods. Each cluster of homologous proteins were then assigned an annotation based on the domain architecture or conserved shared domain. This allowed an initial annotation of gene neighborhoods and their grouping based on conservation of neighborhood associations. The remaining gene neighborhoods were examined for specific template patterns such as TA systems. In this analysis care was taken to ensure that genes are unidirectional on the same strand of DNA and shared a putative common promoter to be counted as a single operon. If they were head to head on opposite strands they were examined for potential bidirectional promoter sharing patterns. We also filtered the data using an intergenic distance criterion of 100 nt for genes to belong to a predicted operon. A complete list of Genbank gene identifiers for proteins investigated in this study is provided in the Additional file 1. TM segments were detected using the TMHMM version 2 program [156] and signal peptides and protein localization were predicted using the Phobius program [157]. Structure similarity searches were conducting using the DALIlite program [158] and structural alignments were generated by means of the MUSTANG program [159].

## Reviewers’ comments

### Reviewer 1: Igor Zhulin

This is a strong, encyclopedic survey and analysis of a large and diverse family of important protein domain families. The search strategy was quite clever. Dealing with remote homologs is never easy and the authors did an excellent job in finding them and then proving their relatedness using extensive profile-profile comparisons and structural considerations. The results lay foundation for future experimental studies in this area, especially when existing domain models in public databases will be appropriately changed. I have decided not to list minor technical points, especially because 50 pages without line and page numbering are hard on a reviewer, and I have only a couple of suggestions to offer:

1. The title sounds as the authors have just discovered the HEPN domain, which is obviously not the case. There should be something in the title (e.g. “survey of”, “redefining”, “novel functions associated with” ) that correctly states their findings.

Response:* We have changed the title as suggested.*

2. Better placement of the new results into the Pfam context would be helpful. HEPN is currently listed as a domain (PF05168) within the nucleotidyltransferase substrate- binding domain clan (KNTase_C, CL0291), not HEPN clan, as stated by authors.

Response:* Yes this is indeed the case. We have now corrected the text to reflect the Pfam nomenclature accurately.*

Authors indicated that their searches retrieved proteins that belong to three families of this clan in addition to HEPN: DUF4145, DUF86 and “C-terminal domains of several polymerase β–superfamily proteins”. The Pfam clan also includes GlnD_UR_UTase, NTase_sub_bind, PaREP1, and DUF294_C domain families. Their relationship to authors’ results remains unclear: are they all “C-terminal domains of several polymerase β–superfamily proteins”? Regardless of the answer, this relationship should be better explained.

Response:* The Pfam models labeled GlnD_UR_UTase, NTase_sub_bind and DUF294_C are C-terminal domains of the polymerase β–superfamily. We have now modified the text to state clear which Pfam models correspond to the several polymerase β–superfamily proteins we mention in the text. In Table *1* we provide a complete mapping of the families to Pfam models. This provides the relationship to our results in terms of new families which we identified.*

### Reviewer 2: Martijn Huynen

The authors present a very comprehensive analysis of the HPEN domain. I appreciate the range of the conclusions that the authors arrive at, from molecular function to evolutionary patterns. The multiplicity of observations and predictions make it very hard to come up with any constructive criticism. Most of the predictions are short stories that may well be true, but about which I do not have enough knowledge to comment upon. So I will only make some short, specific remarks.

Methodological: I cannot really find how the authors decided whether genes are in the same operon, e.g. for the operons depicted in Figure 5. Is there operon information available for all these species? If the operons are inferred based on gene proximity, maybe that should be stated.

Response:* We have now added details regarding how this was done in the Methods section. Briefly, we used two criteria: 1) proximity and 2) conservation of gene-neighborhoods across phylogenetically distant groups of prokaryotes. This data was extracted for each genome using the RefSeq database when possible or the whole-genome-shotgun sequence when the respective genome was missing in RefSeq. We used the gene annotations supplied by the sequencing centers to determine the intergenic distances.*

References: References 64 and 65 are not original references to genes occurring with conserved gene order, or in operons, having related functions.

Response:* We have now added appropriate references to the original works that describe the method of conserved gene-neighborhoods in contextual inference.*

Figures: showing both Figure 3A and B appears redundant.

Response:* We still believe that displaying both depictions has some value because the first reflect the “natural” clustering based on the Kamada-Kawai algorithm, whereas the second is the knowledge-based manual clustering of functions. Showing both helps highlight the fact that there is considerable contextual information that allows discernment of these functional categories from the data itself.*

If the authors want the reader to see the circular permutation in the KEN domain they may have to show it better. To me it is not obvious.

Response:* We have now modified the figure with the N- and C- terminus labeled in order to depict the permutation clearly.*

“The conserved acidic residue” (page 9) is, I take it, part of the second motif mentioned earlier. Please repeat the name of that motif so that the reader knows what is meant”

Response:* We have modified the sentence to clarify the same*

All other editorial changes which were suggested were made.

### Reviewer 3: Nick Grishin

Did not provide any comments.

## Competing interests

The authors declare that they have no competing interests.

## Authors’ contributions

KM, VA, AMB, and LA collected data; KM, VA, EVK and LA analyzed the data; LA and EVK wrote the manuscript that was read and approved by all authors.

## Supplementary Material

Additional file 1 **Provides access to: 1) comprehensive list of Genbank identifiers, architectures and operons and table of HHpred search probability percentages.** 2) A comprehensive raw alignment of HEPN domains. 3) NTD domains combined with HEPN and other defense related domains. 4) Associated domains described for the first time in this work. Click here for file
