# Supplementary material for: Comprehensive analysis of the HEPN superfamily: identification of novel roles in intra-genomic conflicts, defense, pathogenesis and RNA processing
Source: Biol Direct. 2013 Jun 15;8:15. doi: 10.1186/1745-6150-8-15 (PMC3710099; doi:10.1186/1745-6150-8-15)
Supplement: Additional file 1 — Provides access to: 1) comprehensive list of Genbank identifiers, architectures and operons and table of HHpred search probability percentages. 2) A comprehensive raw alignment of HEPN domains. 3) NTD domains combined with HEPN and other defense related domains. 4) Associated domains described for the first time in this work. [file 1745-6150-8-15-S1.html]

HEPN


# 

**HEPN:
A
major nucleic-acid targeting domain involved in intra-genomic
conflicts,
defense, pathogenesis and
RNA processing**

***Vivek
Anantharaman#,
Kira S. Makarova#,
A. Maxwell Burroughs,
Eugene V. Koonin, L. Aravind\****

***National
Center for Biotechnology Information, National
Library of Medicine,   
National
Institutes of Health, Bethesda,
MD
20894, USA  
#******These
authors
made equal contribution to the
work  
\*
Correspondence to: aravind@ncbi.nlm.nih.gov***

  


---

## ABSTRACT

  

**Background:**
The major role of enzymatic toxins that target nucleic acids in
biological
conflicts at all levels has become increasingly apparent thanks in
large part to
the advances of comparative genomics. Typically, toxins evolve fast
which
hampers the identification of these proteins by sequence analysis. Here
we
analyze and unexpectedly widespread superfamily of toxin domains most
of which
possess RNase activity.

**Results:**
The HEPN superfamily is comprised of all α-helical domains
that were first
identified as being associated with DNA polymerase β-type
nucleotidyltransferases in prokaryotes and animal Sacsin proteins.
Using
sensitive sequence and structure comparison methods methods, we vastly
extend
the HEPN superfamily by identifying numerous novel families and by
detecting
diverged HEPN domains in several known protein families. The new HEPN
families include
the RNase LS and LsoA catalytic domains, KEN domains (e.g. RNaseL and
Ire1) and
the RNase domains of RloC and PrrC.  The
majority of HEPN domains contain conserved motifs that constitute a
metal-independent endoRNase active site. The HEPN domains lacking this
motif
are predicted to function as non-catalytic RNA-binding domains, such as
in the
case of the mannitol repressor MtlR. Our analysis shows that HEPN
domains
function as toxins that are shared by numerous systems implicated in
intra-genomic, inter-genomic and intra-organismal conflicts across the
three domains
of cellular life. In prokaryotes HEPN domains are essential components
of
numerous toxin-antitoxin (TA) and abortive infection (Abi) and in
addition are
tightly associated with many restriction-modification (RM) and
CRISPR-Cas
systems, and occasionally with other defense systems such as Pgl and
Ter. We
present evidence of multiple modes of action of HEPN domains in these
system
which include direct attack on viral RNAs (e.g. LsoA and RNase LS) in
conjunction with other RNase domains (e.g. a novel RNase H fold domain,
NamA),
suicidal or dormancy-inducing attack on self RNAs (RM systems and
possibly CRISPR-Cas
systems), and suicidal attack coupled with direct interaction with
phage
components (Abi systems).These findings are compatible with the
hypothesis on
coupling of pathogen-targeting (immunity) and self-directed(programmed
cell
death and dormancy induction) responses in the evolution of robust
antiviral
strategies. We propose that altruistic cell suicide mediated by HEPN
domains
and other functionally similar RNases was essential for the evolution
of kin
and group selection and cell cooperation. The HEPN domains were
repeatedly
acquired by eukaryotes and incorporated into several core functions
such as
endonucleolytic processing of the 5.8S-25S/28S rRNA precursor (Las1), a
novel
ER membrane-associated RNA degradation system (C6orf70), and sensing of
unprocessed transcripts at the nuclear periphery (Swt1). Multiple lines
of
evidence suggest that similar to prokaryotes, HEPN nucleases were
recruited to defense
or apoptotic systems in several groups of eukaryotes.

**Conclusions**: Extensive
sequence and structure
comparisons reveal unexpectedly broad presence of the HEPN domain in an
enormous variety of defense and stress response systems primarily in
prokaryotes but apparently in eukaryotes as well. In addition, HEPN
domains
have been recruited to perform essential functions, in particular in
eukaryotic
rRNA processing. These findings are expected to stimulate experiments
that could
shed light on diverse cellular processes across the three domains of
life.

.
 
   
  


---

## Contents

       
    1) HEPN
familes with gis, operons and architecture; HHpred probability
percentages; domain names   
         
  2) Multiple Alignment of Novel N terminal domains 
       
      
       
    3) Comprehensive Multiple
alignment of HEPN  
            4) Multiple
Alignments of other Novel domains described in the paper  
    
Click here
for the latest version of this file     
      
